# Unraveling the coordination structure-performance relationship in Pt_1_/Fe_2_O_3_ single-atom catalyst

**DOI:** 10.1038/s41467-019-12459-0

**Published:** 2019-10-03

**Authors:** Yujing Ren, Yan Tang, Leilei Zhang, Xiaoyan Liu, Lin Li, Shu Miao, Dang Sheng Su, Aiqin Wang, Jun Li, Tao Zhang

**Affiliations:** 1 State Key Laboratory of Catalysis, Dalian Institute of Chemical Physics, Chinese Academy of Sciences, 116023 Dalian, China; 2 University of Chinese Academy of Sciences, 100049 Beijing, China; 3 Department of Chemistry and Key Laboratory of Organic Optoelectronics and Molecular Engineering of the Ministry of Education, Tsinghua University, 100084 Beijing, China; 4 Department of Chemistry, Southern University of Science and Technology, 518055 Shenzhen, China

**Keywords:** Catalyst synthesis, Heterogeneous catalysis, Density functional theory

## Abstract

Heterogeneous single-atom catalyst (SAC) opens a unique entry to establishing structure–performance relationship at the molecular level similar to that in homogeneous catalysis. The challenge lies in manipulating the coordination chemistry of single atoms without changing single-atom dispersion. Here, we develop an efficient synthetic method for SACs by using ethanediamine to chelate Pt cations and then removing the ethanediamine by a rapid thermal treatment (RTT) in inert atmosphere. The coordination chemistry of Pt single atoms on a Fe_2_O_3_ support is finely tuned by merely adjusting the RTT temperature. With the decrease in Pt-O coordination number, the oxidation state of Pt decreases, and consequently the hydrogenation activity increases to a record level without loss of chemoselectivity. The tunability of the local coordination chemistry, oxidation states of the metal, and the catalytic performance of single atoms reveals the unique role of SACs as a bridge between heterogeneous and homogeneous catalysis.

## Abstract (web summary)

In single-atom catalysts (SACs), little is known about how the coordination structure of single atoms affects the catalytic performance. Here, the authors discover that in Pt_1_/Fe_2_O_3_ SAC, the coordination number of the first shell Pt-O is well correlated with the Pt electronic property and the hydrogenation activity.

## Introduction

As a key fundamental issue in heterogeneous catalysis, structure–performance relationship has been pursued for a long time, yet it remains elusive for a majority of reactions, even for the simple CO oxidation^1–5^. The challenge not only arises from the complicated reactant–catalyst interactions, it also lies in the non-uniform and dynamic structure of the active sites^6,7^. For a supported metal catalyst, the metal particle size and shape, and the support nature, as well as the metal–support interaction, are all possible factors affecting the catalytic performance, and disentangling these interplaying factors to rationalize the structure–performance relationship is not a trivial task even with the high-resolution spectroscopy and microscopy techniques^8–12^. Recently, the advent of single-atom catalyst (SAC) appears as a unique avenue to the molecular level understanding of the coordination structure–performance relationship^13–15^.

SACs are special types of supported metal catalysts in which the active metal species are exclusively dispersed as isolated single atoms via bonding chemically with the surrounding atoms from the support^16^. The extreme single-atom dispersion enables the maximized utility of noble metals that are scarce yet indispensable in applications of energy and environmental catalysis, and the geometric isolation of metal species offers unrivaled selectivity and sometimes even superior activity to the nanoparticle (NP) counterparts for a certain number of reactions^17–24^. In fundamental science, the well-defined mononuclear metal-active site of SACs resembles inorganic or organometallic complexes used as homogeneous catalysts, for which SAC is envisioned to bridge heterogeneous and homogeneous catalysis^25–27^.

Nevertheless, how to build this long-sought bridge relies on finding a key descriptor to establish the structure–performance relationship that works in both homogeneous and heterogeneous catalysis. It has been well documented that the catalytic activity/selectivity of molecularly defined organometallic catalysts can be tuned by modifying organic ligands^28^. In case that the local coordination environment of heterogeneously anchored single atoms is viewed as a rigid ligand embedded in the catalyst surface, the catalytic performance of SACs should be tailored by regulating this environment, just like what has been done in organometallic catalysts. Along this way some long-standing issues in heterogeneous catalysis, such as the role of oxidation state variation at the active site, support effects on the reactivity of metal species, and the nature of the interfacial sites, can hopefully be resolved with fundamental understanding at the atomic level.

Given that the metal single atoms are stabilized by covalent and ionic bonding with the support (e.g., with strong covalent metal–support interaction^29^), the geometric and electronic properties of single atoms should be predominantly determined by the local coordination chemistry^30^. Therefore, we assume that the coordination number (CN) of the metal single atoms might be a reasonable descriptor to rationalize the structure–performance relationship in SACs. In fact, when the metal single atoms are stabilized by N-doped carbon materials, the metal-N CN is shown as a key parameter dictating the catalytic activities^31–36^. For example, medium-spin five coordinated Fe-N5 was several times more active than the other Fe-N*x* species in the selective oxidation of ethylbenzene^32^, and Co-N2 was much more active than Co-N4 for electroreduction of CO_2_ to CO^33^.

In spite of the tunability of N-doped carbons as a host for single metal atoms, the overly strong bonding between metal cations and the N donor limits their activities in certain reactions as evidenced by the high oxidation state of metal species^31–36^. Moreover, N donor is often used as a poison to metal catalysts. Considering that SAC is an extreme regime downsized from metal NPs, it should inherit from some intrinsic properties of metal NPs (e.g., high hydrogenation activity), which is of particular importance to expensive noble metal catalysts. It is therefore highly desired to develop a method for fine-tuning the coordination chemistry of metal single atoms supported on more commonly applied carriers such as oxides. However, for oxide-supported SACs, the metal single atoms are stabilized by coordinating with oxo donors of the support, and the resulting M-Ox structure can be even more rigid than M-Nx due to the robust crystalline structure of the oxide support. It thus poses a great challenge on controlling the coordination chemistry of metal single atoms without destructing the single-atom dispersion.

Herein, we develop an efficient method, abbreviated as en-M-RTT, by using ethanediamine (en) as the ligand to chelate the Pt cations and then removing the ligand by a rapid thermal treatment (RTT) in inert atmosphere. This method not only ensures single-atom dispersion of Pt at a relatively high Pt loading (~1 wt%) on both reducible and non-reducible supports but also allows for fine-tuning the coordination chemistry of Pt single atoms by merely adjusting the RTT temperature. By this approach, we are able to establish the coordination structure–performance relationship by correlating the hydrogenation activity with the average oxidation state of Pt and the CN of Pt-O.

## Results

### Synthesis and characterization of Pt_1_/Fe_2_O_3_-T catalysts

**Fig. 1:**
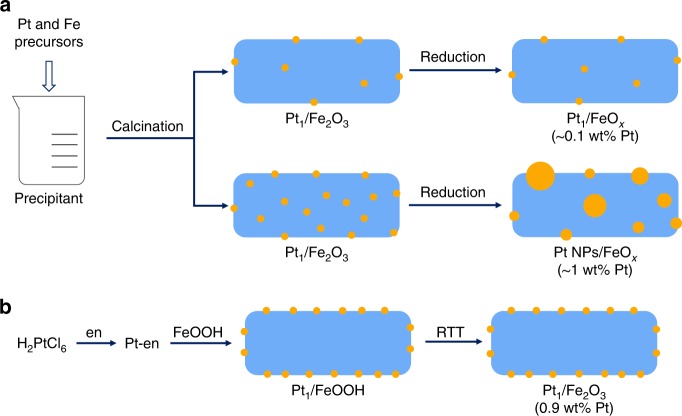
Schematic illustration of different synthesis methods for Pt_1_/Fe_2_O_3_ catalysts. **a** Co-precipitation (CP) method, showing the possible burying of a portion of Pt single atoms inside the Fe_2_O_3_ support and the aggregation of single atoms during post-activation (reduction) process in the case of relatively high Pt loadings. **b** en-M-RTT method, showing the relatively high density of Pt single atoms predominantly located on the Fe_2_O_3_ surface

The iron oxide-supported Pt SACs are usually prepared with a co-precipitation (CP) method followed by a reduction activation procedure^13,23^ (Fig. 1a); however, the CP method suffers from the imbedding of a portion of Pt single atoms inside the iron oxide, thus decreasing the accessibility of active Pt species, while the reduction activation procedure tends to cause the aggregation of single atoms, for which the Pt metal loading must be kept pretty low (~0.1 wt%) to ensure the single-atom dispersion. Owing to the tendency to aggregation, this conventional preparation approach precludes the tuning of coordination chemistry of single atoms. In order to overcome these limitations, here we explore an efficient method (en-M-RTT, Fig. 1b) by using ethanediamine (en) as the ligand to chelate the Pt cations and then removing the ligand by a RTT in He. It was reported earlier that the en-ligand favored dispersion of nickel as isolated divalent Ni^II^ on silica support^37^, while the RTT offered unique advantage in removing the organic ligand without significant sintering of metal species^38^. Taking it into account that the extremely high heating and cooling rates (Supplementary Fig. 1) of RTT will favor the kinetic transformations over thermodynamics, we expect that this unique en-M-RTT method will allow for fine-tuning the coordination chemistry of SACs.

**Fig. 2:**
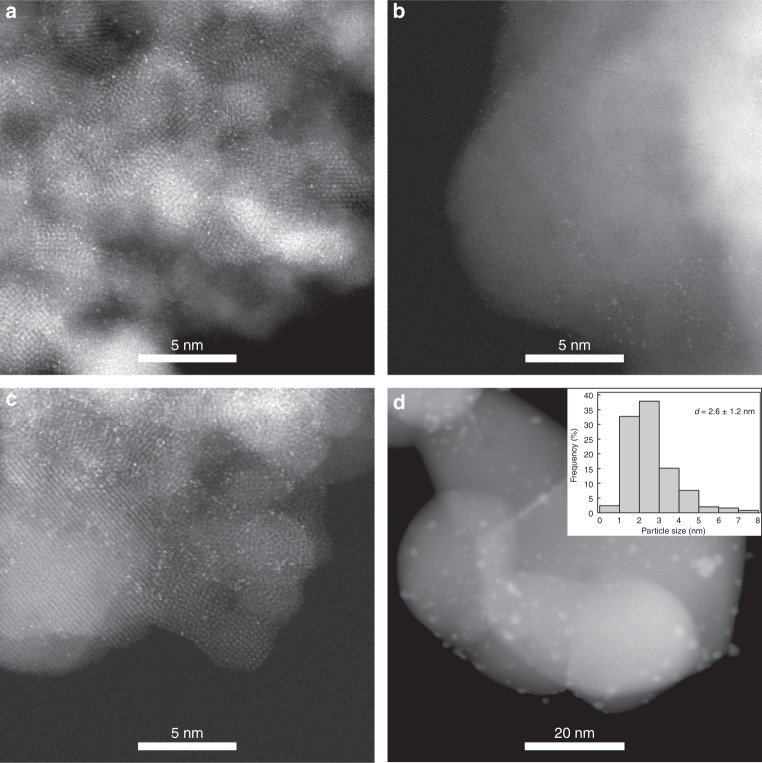
HAADF-STEM images of Pt_1_/Fe_2_O_3_-T SACs and Pt/Fe_2_O_3_ NP catalyst. A large number of single atoms of Pt (bright dots) are clearly observed in Pt_1_/Fe_2_O_3_-500 **a**, Pt_1_/Fe_2_O_3_-550 **b**, and Pt_1_/Fe_2_O_3_-600 **c** catalysts, while non-uniform NPs are observed in Pt/Fe_2_O_3_-600-10 min **d** NP catalyst (the inset is the histogram of the Pt particle size distribution)

By varying the RTT temperature from 500 to 600 °C while keeping the RTT time for only 1 min, we could obtain a series of Pt_1_/Fe_2_O_3_-T (T denotes the RTT temperature) SACs. Figure 2 and Supplementary Fig. 2 show the representative aberration-corrected high-angle annular dark-field scanning transmission electron microscopy (AC-HAADF-STEM) images, which clearly reveal the single-atom dispersion for Pt_1_/Fe_2_O_3_-500, Pt_1_/Fe_2_O_3_-550, and Pt_1_/Fe_2_O_3_-600 catalysts. However, at a longer pyrolysis time, for example, at 600 °C for 10 min, significant aggregation took place resulting in the formation of Pt NPs in the range of 1–5 nm (Fig. 2d), demonstrating the unique advantage of RTT in preventing the metal single atoms from aggregation. Consistent with the HAADF-STEM observations, the X-ray diffraction (XRD) patterns of the series of Pt_1_/Fe_2_O_3_-T catalysts showed only Fe_2_O_3_ crystalline phase without any Pt or Pt-oxide phase (Supplementary Fig. 3), indicating all the Pt species is highly dispersed. To be noted, all these Pt_1_/Fe_2_O_3_-T SACs have relatively high Pt loadings (0.9 wt%, Supplementary Table 1), which could not be achieved with conventional deposition-reduction method. In addition to RTT, the use of en-ligand was also a key factor in maintaining the single-atom dispersion of Pt species. The control experiments without the en-ligand or with ethylene glycol to replace en all led to the formation of Pt NPs (Supplementary Figs. 4, 5), although the same RTT program was used for post-activation. This result underlines the superiority of combining the en-ligand with the RTT post-activation procedure. We further explored the application of en-M-RTT method to other supported SACs with high Pt loadings, and succeeded in both reducible and non-reducible supported Pt SACs (Supplementary Figs. 6, 7). The power of the en-M-RTT method manifested in the controllable preparation of high-loading supported SACs deems to provide a unique entry to fine-tuning the local coordination chemistry of the central single atoms of SACs.

### Coordination and electronic structure

**Fig. 3:**
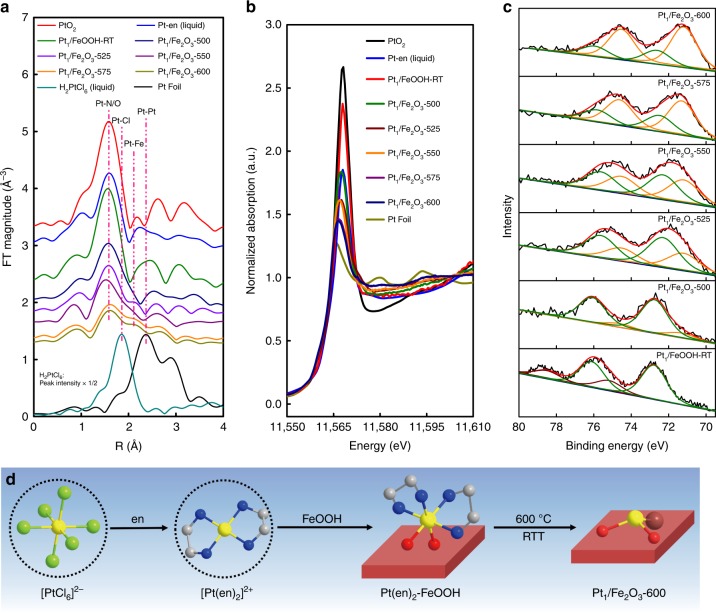
The Pt L_III_-edge XAS and Pt 4*f* XPS data of the Pt_1_/Fe_2_O_3_-T catalysts. **a** The Fourier-transformed *k*^2^-weighted EXAFS spectra in R-space (without phase correction), **b** the normalized XANES spectra, **c** the Pt 4*f* XPS spectra, and **d** schematic representation of the structure evolution of Pt species in different stages of preparation. Color codes: yellow (Pt), green (Cl), blue (N), gray (C), red (O), and brown (Fe)

Since the series of the Pt_1_/Fe_2_O_3_-T SACs all maintain the single-atom dispersion, while the local structure of the Pt single atoms depends on the RTT temperature (*T*), we subsequently probed the local coordination chemistry of the Pt single atoms by jointly using X-ray absorption spectroscopy (XAS) and X-ray photoelectron spectroscopy (XPS) techniques. In the Pt L_III_-edge X-ray absorption fine structure (EXAFS) spectra (Fig. 3a), all the Pt_1_/Fe_2_O_3_-T samples show a major peak at a distance of 1.6–1.8 Å, which can be ascribed to the coordination of Pt atom to light elements like oxygen or nitrogen. The continuous decrease in the peak intensity with increasing the RTT temperature from RT to 600 °C reflects a gradual decrease in the CN of Pt to O (or N). On the other hand, at the distance of Pt–Pt coordination (with Pt foil as the reference) there is no peak for the series of Pt_1_/Fe_2_O_3_-T (T: 500–600) catalysts, corroborating the single-atom dispersion in all of these samples. However, for the sample Pt_1_/FeOOH-RT, a broad peak appears at ~ 2.4 Å, which can be reliably ascribed to the collective contributions from both Pt-C and Pt-Fe (Pt-O-Fe) coordination in the second shell, as illustrated in Fig. 3d. The Pt-C coordination in the second shell arises from the [Pt(en)_2_]^2+^ complex, which can be approved by comparison with the precursor [Pt(en)_2_]^2+^. However, since the Pt-C is in the second shell and it is difficult to obtain a reliable fitting result, it will not be considered in the following fitting. The best-fitted result in the first shell is shown in Table 1. In detail, the [Pt(en)_2_]^2+^ precursor shows a Pt-N coordination at 2.01 Å with CN of 4.3. When this precursor is electrostatically adsorbed on the FeOOH support, it is oxidized by the FeOOH support as evidenced by the remarkable increase of the white-line intensity in the X-ray absorption near edge structure (XANES) (Fig. 3b) as well as the increased CN of Pt-N/O from 4.3 to 6.0. At this stage, each isolated Pt cation coordinates with four nitrogen atoms from the en-ligand and two oxygen atoms from the FeOOH support (Fig. 3d). With RTT process, the en-ligand was decomposed and the Pt cation was coordinated with the Fe_2_O_3_ support. Moreover, the CN of Pt-O in the first shell continuously decreased from 3.8 to 1.8 when the RTT temperature was raised from 500 to 600 °C, and the Pt-Fe coordination began to appear at *T* ≥ 525 °C, although its contribution was relatively small. Concurrently, the white-line intensity gradually decreased, indicating the oxidation state of Pt became lower. As such, the coordination and oxidation state of Pt single atoms can be successfully tuned by merely changing the RTT temperature.

**Table 1:** The best-fitted EXAFS results of Pt_1_/Fe_2_O_3_-T catalysts^a^

Sample	Shell	CN	*R* (Å)	*σ*^2^ (10^−2^ Å^2^)	Δ*E*_0_ (eV)	*r*-factor (%)
Pt Foil	Pt-Pt	12.0	2.76	0.5	4.9	0.001
PtO_2_	Pt-O	6.0	1.99	0.4	3.2	0.3
H_2_PtCl_6_ (liquid)	Pt-Cl	6.0	2.32	0.4	−3.7	0.02
Pt-en (liquid)	Pt-N	4.3	2.01	0.5	5.7	0.3
Pt_1_/FeOOH-RT	Pt-(N/O)	6.0	1.99	0.6	0.3	0.5
Pt_1_/Fe_2_O_3_-500	Pt-O	3.8	2.00	0.7	4.5	0.6
Pt_1_/Fe_2_O_3_-525	Pt-O	3.1	2.00	0.7	5.1	0.6
	Pt-Fe	0.2	2.63	0.4	5	
Pt_1_/Fe_2_O_3_-550	Pt-O	2.8	2.00	0.7	4.9	0.6
	Pt-Fe	0.3	2.61	0.3	4.9	
Pt_1_/Fe_2_O_3_-575	Pt-O	2.0	2.02	0.6	7	0.2
	Pt-Fe	0.7	2.62	0.3	7	
Pt_1_/Fe_2_O_3_-600	Pt-O	1.8	2.02	0.6	6.5	0.5
	Pt-Fe	0.8	2.63	0.3	6.5	

^a^CN is the coordination number for the absorber–backscatterer pair, *R* is the average absorber−backscatterer distance, *σ*^2^ is the Debye−Waller factor, and Δ*E*_0_ the inner potential correction. The accuracies of the above parameters are estimated as CN, ±20%; *R*, ±1%; *σ*^2^, ± 20%; Δ*E*_0_, ±20%. The data range used for data fitting in *k*-space (Δ*k*) and *R*-space (Δ*R*) are 3.0–10.5 Å^−1^ and 1.2–3.2 Å, respectively

In order to quantify the average oxidation state of Pt single atoms in different Pt_1_/Fe_2_O_3_-T catalysts, we integrate the white-line area and correlate it to the average oxidation state of Pt by using Pt foil and PtO_2_ as the references (details are given in the Supplementary Table 1). The result is summarized in Supplementary Table 1. It can be seen that the average oxidation state of Pt in the catalyst precursor (Pt_1_/FeOOH-RT) is 2.46, and it continuously decreases with increasing the RTT temperature until 0.56 for the Pt_1_/Fe_2_O_3_-600. In line with this result, the XPS analysis also shows that the oxidation state of Pt decreases with increasing the RTT temperature, as indicated by the gradual shift of the Pt 4*f* binding energy to lower values (Fig. 3c). Further peak deconvolution and integration result reveal that the catalyst precursor (Pt_1_/FeOOH-RT) containing 24.0% Pt^IV^ and 76.0% Pt^II^ is transformed into more reduced state upon RTT; for example, the Pt_1_/Fe_2_O_3_-600 is composed of 25.7% Pt^II^ and 74.3% Pt^0^ (Supplementary Table 1). In other words, the average oxidation state of Pt for the Pt_1_/Fe_2_O_3_-600 was 0.51, slightly lower than that determined by XANES. Moreover, the surface Pt/Fe atomic ratio in all the Pt_1_/Fe_2_O_3_-T catalyst are several times higher than the bulk value of 0.37% (Supplementary Table 1), suggesting that the Pt single atoms are predominantly on the surface without being buried in the support, which is beneficial to the surface catalysis.

### Catalytic performance

Now we have shown that the coordination and electronic structures of the Pt single atoms could be fine-tuned by merely changing the RTT temperature. Then we seek how the coordination and electronic structures affect the catalytic performance. Here we choose the chemoselective reduction of 3-nitrostyrene as a probe reaction to establish the correlation between structure and performance. Table 2 summarizes the reaction data of the series of Pt_1_/Fe_2_O_3_-T catalysts. It is noted that neither pure Fe_2_O_3_ support nor the Pt_1_/FeOOH-RT was active at all for the reaction. By contrast, upon being activated by RTT procedure, all the Pt_1_/Fe_2_O_3_-T SACs exhibited pronounced activity and excellent chemoselectivity, indicating that the under-coordinated Pt single atoms are the active center. Moreover, the reaction time required to achieve the conversion >95% decreased significantly with the RTT temperature; meanwhile, the chemoselectivity all remained at a very high value (95–98%) even at conversions close to 100%, which is the unique feature of Pt SACs based on our previous studies^23,39^. The turnover frequency (TOF) is calculated based on the total Pt loading because all of the Pt single atoms are on the surface and accessible to reactants, and it increases remarkably with the RTT temperature. Among the series of the Pt_1_/Fe_2_O_3_-T catalysts, the Pt_1_/Fe_2_O_3_-600 is the most active, affording a TOF of 3809 h^−1^ at the reaction temperature of 40 °C and as high as 21,099 h^−1^ at 60 °C, which is at least one order of magnitude more active than those noble metal NP catalysts reported earlier^40–42^ and references in Supplementary Table 2, and is also 2–3 times more active than our earlier reported Pt_1_/FeO_*x*_ SACs (Supplementary Table 2)^23,39^. Here it should be underscored that such a record-high activity is achieved with a high Pt-loading catalyst, meaning that the catalyst is also able to afford a rather high efficiency even based on the total weight of catalyst (111 kg_product_ h^−1^ kg_cat_^−1^), which is of practical importance yet difficult to achieve by those very dilute SACs. Meanwhile, we must emphasize that the single-atom dispersion is the most important factor dictating the chemoselectivity. For example, the Pt/Fe_2_O_3_-600-10 catalyst, which is composed mainly of Pt NPs, gives rise to a selectivity of only 63.1%. The uniqueness of the Pt SACs for high chemoselectivity arises from the unique adsorption mode^24^. When the Pt species is dispersed as single atoms, the substrate will adsorb via a top mode, that is, the nitro group is preferentially adsorbed through its two oxygen atoms on Pt single atom and the neighboring Fe atom^24^ or oxygen vacancy^42,43^, thus leading to high chemoselectivity. In contrast, upon formation of Pt NPs, the substrate will be adsorbed on Pt NPs in a lying mode, that is, both the –C = C group and the benzene ring can be adsorbed on the catalyst surface, thus leading to the hydrogenation of –C = C bond, giving rise to low chemoselectivity.

**Table 2:** Chemoselective hydrogenation of 3-nitrostyrene over Pt_1_/Fe_2_O_3_-T catalysts

Catalyst	Time (min)	Conv. (%)	Sel. (%)	TOF (mol_conv._ h^−1^ mol_Pt_^−1^)
Fe_2_O_3_	240	–	–	–
Pt_1_/FeOOH-RT	300	–	–	–
Pt_1_/Fe_2_O_3_-500	140	95.6	95.1	491
Pt_1_/Fe_2_O_3_-525	50	96.4	98.1	1411
Pt_1_/Fe_2_O_3_-550	45	95.6	97.1	1531
Pt_1_/Fe_2_O_3_-575	20	97.3	96.2	3346
Pt_1_/Fe_2_O_3_-600	18	98.9	98.0	3809
Pt_1_/Fe_2_O_3_-600^a^	6	97.2	96.2	21,099
Pt/Fe_2_O_3_-600-10 min	23	97.8	63.1	–

Pre-treatment condition: 5 mL toluene, 10 bar H_2_, 40 °C, 1 h. Reaction conditions: 40 °C, 3 bar H_2_, 10 mg catalyst and 90 mg Fe_2_O_3_. Five-milliliter reaction mixture: 0.5 mmol substrate, toluene as solvent, *o*-xylene as internal standard

^a^60 °C, 10 bar H_2_, 5 mg catalyst, and 95 mg Fe_2_O_3_

Given that the RTT in a very short time may yield a thermodynamically unstable phase, one would question if this state can be maintained during the hydrogenation reaction. To address this question, we investigated the reaction stability of the Pt_1_/Fe_2_O_3_-600 catalyst. After five reuse tests, the activity decreased only slightly, whereas the selectivity remained unchanged (Supplementary Table 3). The slight decay in activity was caused by the adsorption of organic compounds, which was confirmed by a clear weight loss of 1.5 wt% at 250 °C in the thermogravimetric analysis profile of the spent catalyst (Supplementary Fig. 8). Further characterizations with AC-HAADF-STEM, XPS, XAS, and inductively coupled plasma (ICP) analysis of the spent catalyst (after the fifth cycle) showed that the Pt single atoms remained isolated without any aggregation (Supplementary Fig. 9) or being leached (Supplementary Table 4), and the chemical state of Pt remained essentially unchanged after repetitive reaction tests (Supplementary Figs. 10, 11 and Supplementary Table 4). All these results have demonstrated good stability of the catalyst. Moreover, the catalyst had excellent substrate adaptability (Supplementary Table 5), which holds great promise in practical applications, especially because of the facile synthesis method and relatively high loading of Pt.

### Structure and performance relationship

**Fig. 4:**
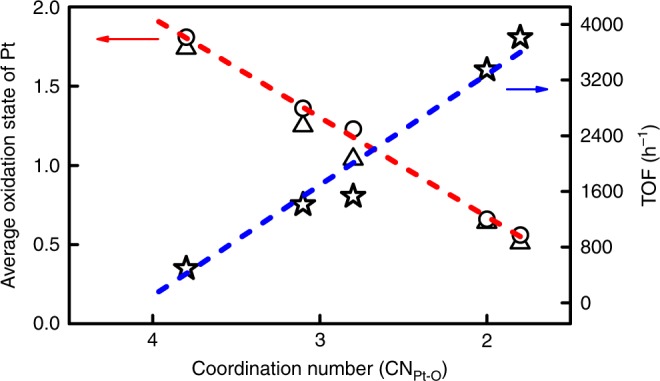
The correlation between coordination structure and catalytic performance. The star, triangle, and circle represent TOF, average oxidation state of Pt determined from XPS and XANES, respectively. Linear correlations are obtained between the CN of Pt-O and the average oxidation state (red line), and between CN of Pt-O and the hydrogenation activity (blue line)

Figure 4 illustrates the correlation between catalytic activity and coordination structure, with CN (determined by EXAFS) of Pt in the first shell Pt-O as *x*-axis while average oxidation state of Pt (determined by both XANES and XPS) as one *y*-axis and TOF as the other *y-*axis. It is interesting that with a decrease of the Pt-O CN, the oxidation state of Pt single atoms decreases in an almost linear correlation, suggesting that the electronic structure is largely determined by the local coordination chemistry of single atoms with the surrounding donor atoms from the support, resembling the metal–ligand interaction in organometallic complexes^44^. More interestingly, when further correlating with the catalytic activity (TOF), we got approximately linear relationship between them, that is, the smaller CN of Pt-O, the lower oxidation state of Pt single atoms, the higher catalytic activity. Such a structure–activity relationship well demonstrates that the catalytic performance of SAC can be fine-tuned and optimized via altering the local coordination environment, just like modifying the ligand of homogeneous organometallic catalysts. Similar to our findings, Wan and co-workers^45^ reported that the maximum *d*-charge gain was obtained on the Pd single atom in the Pd-Au nanoalloy, which led to enhanced activity. In this sense, SAC is a natural bridge between homogeneous and heterogeneous catalysis^25–27^. On the other hand, the almost linear dependence of catalytic activity on the coordination chemistry of Pt-O strongly suggests that the active site in the Pt_1_/Fe_2_O_3_ SACs is the (Fe)_*n*_-Pt_1_-(O-Fe)_*m*_ ensemble, with Pt single atom at the core. We suppose that the metal–ligand ensemble active site holds true for other supported SAC systems.

To understand how this low Pt-O coordination environment promotes the catalytic reaction, we first conducted hydrogen chemisorption experiment with H_2_-microcalorimetry. As shown in Supplementary Fig. 12 and Supplementary Table 6, both the adsorption heat and the H_2_ uptake increased greatly with the RTT temperature (Pt_1_/Fe_2_O_3_-600 > Pt_1_/Fe_2_O_3_-550 > Pt_1_/Fe_2_O_3_-500). That is, with the decrease in CN of Pt-O (1.8 < 2.8 < 3.8), the electron density of Pt single atoms increases with less vacant *d*-states, thus making Pt single atoms closer to metallic Pt and therefore facilitating the adsorption and activation of H_2_ molecules. To determine whether the H_2_ activation is a rate-determining step (RDS) in the chemoselective hydrogenation of 3-nitrostyrene, we then conducted a kinetic isotope effect (KIE) experiment by changing the H_2_ atmosphere to D_2_. Consistent with the activity trend and H_2_-microcalorimetric result, the Pt_1_/Fe_2_O_3_-500 has a much larger KIE than Pt_1_/Fe_2_O_3_-600 (6.55 vs. 2.03, Supplementary Table 7), suggesting that H_2_ activation is more difficult for the Pt_1_/Fe_2_O_3_-500. It is noted that the KIE value obtained on the Pt_1_/Fe_2_O_3_-600 catalyst is comparable to those reported on noble metal NP catalysts^46,47^, suggesting that the surface reaction, rather than the hydrogen dissociation, is the RDS. In contrast, the similar large KIE to the Pt_1_/Fe_2_O_3_-500 was reported for Ag/Al_2_O_3_ NPs as well as Pd_1_/TiO_2_ SAC, both of which featured poor capability for hydrogen activation^19,48^. Therefore, it seems reasonable that the RDS for nitroarene hydrogenation over the Pt_1_/Fe_2_O_3_-500 is the hydrogen dissociation.

### DFT calculations

To gain insights in the details of the coordination chemistry of single Pt atoms in Pt_1_/Fe_2_O_3_-T catalysts, periodic density functional theory (DFT) calculations were carried out. Considering that the Pt_1_/FeOOH-RT was inactive at all for the target hydrogenation reaction, we started to model with the Pt_1_/Fe_2_O_3_-500 that has a Pt-O CN of 4 (Table 1). The Pt_1_/Fe_2_O_3_-500 model was established by supporting one Pt atom on Fe_2_O_3_(0001) surface (Supplementary Fig. 13a). After optimization, the single-atom Pt coordinates with four surface oxygen atoms in a distorted square geometry with an average Pt-O length of 1.94 Å, which is in good agreement with EXAFS data. Bader charge analysis indicates that the single-atom Pt on Fe_2_O_3_(0001) is positively charged (+1.48 |*e*|). The three-coordinated Pt_1_/Fe_2_O_3_-550 and two-coordinated Pt_1_/Fe_2_O_3_-600 catalysts were modeled by removing one and two surface oxygen atoms, respectively (Supplementary Fig. 13b, c). For the two-coordinated Pt_1_/Fe_2_O_3_-600 catalyst, one Fe-Pt bond forms with the bond length of 2.68 Å, consistent well with the experimental results. The Bader charge of the single-atom Pt in the three-coordinated Pt_1_/Fe_2_O_3_-550 catalyst is +1.31 |*e*|, which is lower than that in the four-coordinated Pt_1_/Fe_2_O_3_-500 catalyst, but larger than that in the two-coordinated Pt_1_/Fe_2_O_3_-600 catalyst (+0.78 |*e*|). These results agree well with the experiments showing that the oxidation state of Pt single atoms decreases with the decrease of the Pt-O CN.

**Fig. 5:**
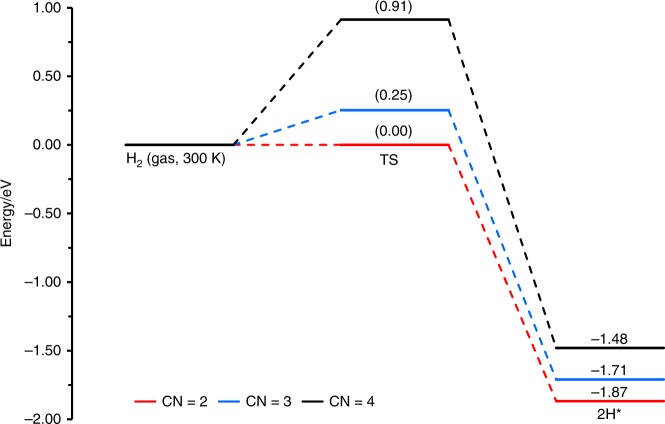
Energy profiles of H_2_ dissociation on Pt_1_/Fe_2_O_3_-T catalysts at 300 K. Four-coordinated Pt_1_/Fe_2_O_3_-500 (black line), three-coordinated Pt_1_/Fe_2_O_3_-550 (blue line), and two-coordinated Pt_1_/Fe_2_O_3_-600 (red line) surfaces

The H_2_-dissociated adsorption energies on the three Pt_1_/Fe_2_O_3_-T catalysts with different Pt-O CNs were also calculated. The optimized adsorption structures, as shown in Supplementary Fig. 14, reveal that the adsorption strengths of H_2_ follow the order of four-coordinated Pt_1_/Fe_2_O_3_-500 (*E*_ads_ = −1.80 eV) < three-coordinated Pt_1_/Fe_2_O_3_-550 (*E*_ads_ = −2.03 eV) < two-coordinated Pt_1_/Fe_2_O_3_-600 (*E*_ads_ = −2.19 eV). We further calculated the reaction barrier of H_2_ dissociation for the three catalysts. Figure 5 and Supplementary Fig. 15 show the energy profiles under reaction conditions (300 K) and the corresponding optimized geometric structures of transition states. The barrier of H_2_ dissociation on three-coordinated Pt_1_/Fe_2_O_3_-550 is 0.25 eV, much lower than that on four-coordinated Pt_1_/Fe_2_O_3_-500, indicating that the three-coordinated Pt_1_/Fe_2_O_3_ catalyst is more active for H_2_ activation than the four-coordinated Pt_1_/Fe_2_O_3_-500. In particular, H_2_ can dissociate spontaneously on the two-coordinated Pt_1_/Fe_2_O_3_-600 surface at 300 K. The reaction energies under 300 K for four-, three-, and two-coordinated Pt_1_/Fe_2_O_3_ are −1.48, −1.71, and −1.87 eV, respectively. Hence, with the decrease in the Pt-O CN, it is much easier for Pt single atoms to activate H_2_ molecules, making it several times more active in the hydrogenation reactions. Therefore, the coordination structures control the catalytic performance by virtue of manipulating the oxidation states and local electronic structures of the active center.

## Discussion

Different from NP catalysts, where the particle size and crystal plane often exposes significant effect on the activity/selectivity, catalytic performance of SACs featured with isolated metal single atoms coordinated by support donors is dictated by the local coordination chemistry of single atoms. For the Pt_1_/Fe_2_O_3_ catalyst, we have unequivocally demonstrated that both the average oxidation state of Pt and the hydrogenation activity were linearly correlated with the Pt-O CN in the first shell of Pt single atom, while the ultra-high chemoselectivity was solely determined by the single-atom dispersion. Such a structure–performance relationship mirrors the high similarity between heterogeneous SACs and homogeneous organometallic complexes, and opens an avenue to rational design of catalysts with both high activity and selectivity without being limited by the conventional scaling relationship^49,50^.

## Methods

### Sample preparation

The FeOOH support was prepared by a precipitation method. Briefly, 4 g ammonium carbonate ((NH_4_)_2_CO_3_) was put into 60 mL deionized water and stirred at 50 °C until it was completely dissolved. Then, 20 mL of an aqueous solution of ferric nitrate (Fe(NO_3_)_3_, 1 mol L^−1^) was added and stirred for 3 h. After aging for 3 h at 50 °C, the solid was filtrated and washed with deionized water and finally dried at 60 °C for 12 h. The Brunauer–Emmett–Teller (BET) surface area of the as-prepared FeOOH support was 266 m^2^ g^−1^.

The Pt-en precursor was prepared by mixing 10 mL of an aqueous solution of chloroplatinic acid (H_2_PtCl_6_, 5 mmol L^−1^) with 1 mL ethanediamine (en) at room temperature. Then, this Pt-en precursor was added to a suspension of the FeOOH support (1 g in 10 mL deionized water). After stirring for 4 h, the solid was filtrated and washed with deionized water and finally dried at 60 °C for 12 h to obtain the Pt_1_/FeOOH-RT sample.

The series of Pt_1_/Fe_2_O_3_-T catalysts were prepared by RTT of Pt_1_/FeOOH-RT in He at the specified temperature for 1 min. Briefly, the Pt_1_/FeOOH-RT sample was put into a quartz tube, which was then inserted into a tube furnace pre-heated to the specified temperature. Under the He flow (30 mL min^−1^) the sample was kept at that temperature for 1 min, and then the quartz tube was quickly taken out and rapidly cooled down to RT (Supplementary Fig. 1). The Pt/Fe_2_O_3_-600-10 min NP catalyst was prepared by the same method, except for keeping at 600 °C for 10 min.

More sample preparation details are described in the Supplementary Methods section.

### Sample characterization

The actual Pt loadings were determined by inductively coupled plasma atomic emissionspectroscopy on an IRIS Intrepid II XSP instrument (Thermo Electron Corporation). XRD patterns were recorded on a PANalytical X’ pert diffractometer with a Cu-Kα radiation source (40 kV and 40 mA). A continuous mode was used to record data in the 2*θ* range from 10° to 80°. N_2_ adsorption–desorption experiments were conducted on a Micromeritics ASAP-2010 physical adsorption apparatus. Before the measurement, the sample was pretreated at 110 °C for 12 h in vacuum. The specific surface area was calculated using a BET method.

STEM and energy-dispersive X-ray spectroscopy (EDS) experiments were performed on a JEOL JEM-2100F microscope operated at 200 kV, equipped with an Oxford Instruments ISIS/INCA EDS system with an Oxford Pentafet Ultrathin Window (UTW) Detector. The AC-HAADF-STEM analysis was performed on a JEOL JEM-ARM200F equipped with a CEOS probe corrector, with a guaranteed resolution of 0.08 nm. Before microscopy examination, the sample was ultrasonically dispersed in ethanol for 15–20 min, and then a drop of the suspension was dropped on a copper TEM grid coated with a thin holey carbon film.

The XAS including XANES and extended EXAFS at Pt L_III_-edge of the samples were measured at the beamline 14 W of Shanghai Synchrotron Radiation Facility (SSRF) in China. The output beam was selected by Si(111) monochromator, and the energy was calibrated by Pt foil. Before measurement, the samples were diluted by boron nitride and tableted. The data were collected at room temperature under fluorescence mode by using Lytle detector. Athena software package was employed to process the XAS data.

XPS spectra were obtained on a Thermo ESCALAB 250 X-ray photoelectron spectrometer equipped with Al Kα excitation source and with C as the internal standard (C 1*s* = 284.6 eV).

More characterization performance details are described in the Supplementary Methods section.

### Catalytic testing

The chemoselective hydrogenation of 3-nitrostyrene was performed in an autoclave equipped with Teflon lining. Before the reaction test, the catalyst was pre-reduced in situ by 10 bar hydrogen in toluene at 40 °C for 1 h. Then, the toluene was poured out and a total volume of 5 mL solution including 3-nitrostyrene, toluene, and internal standard *o*-xylene, was put into the autoclave. Afterwards, the autoclave was purged with hydrogen several times and pressurized with hydrogen to 3 bar. The reaction was started by heating the mixture to 40 °C under vigorous stirring at a speed of 800 r.p.m. After the reaction, the product was analyzed by gas chromatography (Agilent 7890B).

The conversion of 3-nitrostyrene and the selectivity of 3-vinylaniline were calculated using (1) and (2):

1 $${\mathrm{Conv}}\;\left( \% \right) = \left( {{\mathrm{mol}}_{{3{\mbox{-}\mathrm{nitrostyrene}}}\;{\mathrm{consumed}}}} \right)/\left( {{\mathrm{mol}}_{3{\mbox{-}\mathrm{nitrostyrene}}\;{\mathrm{fed}}}} \right) \times 100.$$

2 $${\mathrm{Sel}}\;\left( \% \right) = \left( {{\mathrm{mol}}_{{3{\mbox{-}}\mathrm{vinylaniline}}\;{\mathrm{produced}}}} \right)/\left( {{\mathrm{mol}}_{{3{\mbox{-}}\mathrm{nitrostyrene}}\;{\mathrm{consumed}}}} \right)\times 100.$$

The TOF was calculated using (3) when the conversion of 3-nitrostyrene is below 30%, considering that the conversion increased linearly with the reaction time (see Supplementary Fig. 16)

3 $${\mathrm{TOF}} = \left( {{\mathrm{mol}}_{{3{\mbox{-}}\mathrm{nitrostyrene}}\;{\mathrm{consumed}}}} \right)/\left( {\left( {{\mathrm{mol}}_{{\mathrm{Pt}}\;{\mathrm{in}}\;{\mathrm{catalyst}}}} \right) \times \left( {{h}_{{\mathrm{reaction}}\;{\mathrm{time}}}} \right)} \right).$$

### Theoretical approach

All the theoretical calculations were carried out using Kohn–Sham DFT with periodic boundary condition. We modeled the α-Fe_2_O_3_-*p*(4 × 4)(0001) surface with *p*(4 × 4) supercells that have 12 layers of Fe atoms and 7 atomic layers of O_3_ to model the O-terminated surface. The reaction transition states were verified using vibrational frequency analysis. The theoretical and computational details are given in the Supplementary Methods section.

## Supplementary information

Supplementary Information

Peer Review

## Data Availability

All data are available within the article and its Supplementary Information file are available from the authors upon request.
