# Supplementary material for: Unraveling the coordination structure-performance relationship in Pt1/Fe2O3 single-atom catalyst
Source: Nat Commun. 2019 Oct 3;10:4500. doi: 10.1038/s41467-019-12459-0 (PMC6776505; doi:10.1038/s41467-019-12459-0)
Supplement: Supplementary file 1 — Supplementary Information [file 41467_2019_12459_MOESM1_ESM.pdf]

Unravelling the coordination structure-performance relationship in Pt_1/Fe_2O_3 single-atom catalyst

Ren et al.

Supplementary Methods

In the sample preparation section, the FeOOH support was prepared by a precipitation method. Briefly, 4 g ammonium carbonate ($(NH_4)_2CO_3$) was put into 60 ml deionized water and stirred at 50 ^oC until it was completely dissolved. Then, 20 ml of an aqueous solution of ferric nitrate ($Fe(NO_3)_3$, 1 mol L^{-1}) was added and stirred for 3 h. After aging for 3 h at 50 ^oC, the solid was filtrated and washed with deionized water and finally dried at 60 ^oC for 12 h. The BET surface area of the as-prepared FeOOH support was 266 m^2 g^{-1}.

The CeO_2 support was prepared by hydrothermal method. Briefly, 1.736 g cerium nitrate hexahydrate ($Ce(NO_3)_3 \cdot 6H_2O$) and 19.2 g sodium hydroxide (NaOH) were put into 10 and 70 mL deionized water and stirred until they were completely dissolved. Then, the two solutions were mixed and the mixture was stirred for 30 min. After that, the turbid liquid was transferred into a Teflon-lined autoclave and heated at 110 ^oC in an oven for 24 h. After natural cooling down to room temperature, the solid products were collected by filtration, and washed with deionized water, and finally dried at 60 ^oC for 12 h.

The activated carbon (AC) support was prepared by treating Norit AC with concentrated nitric acid solution (33 wt%) at 60 ^oC for 24 h. Then, the solid was collected by filtration, and washed with deionized water several times to neutral, and finally dried at 120 ^oC for 12h.

The Pt-en precursor was prepared by mixing 10 ml of an aqueous solution of chloroplatinic acid (H_2PtCl_6, 5 mmol L^{-1}) with 1 mL ethanediamine (en) at room temperature. Then this Pt-en precursor was added to a suspension of the FeOOH support (1g in 10 mL deionized water). After stirring for 4h, the solid was filtrated and washed with deionized water and finally dried at 60 ^oC for 12 h to obtain the Pt_1/FeOOH-RT sample.

The series of Pt_1/Fe_2O_3-T catalysts were prepared by rapid thermal treatment (RTT) of Pt_1/FeOOH-RT in He at the specified temperature for 1 min. Briefly, the Pt_1/FeOOH-RT sample was put into a quartz tube which was then inserted into a tube furnace pre-heated to the specified temperature. Under the He flow (30 ml min^{-1}) the sample was kept at that temperature for 1 min, then the quartz tube was quickly taken out and rapidly cooled down to RT (Supplementary Figure 1). The Pt/Fe_2O_3-600-10min NPs catalyst was prepared by the same method except for keeping at 600 ^oC for 10 min.

For comparison, four control samples, H_2PtCl_6/Fe_2O_3-600, H_2PtCl_6-ethylene glycol/Fe_2O_3-

600, Pt_1/CeO_2-600 and Pt_1/AC-600 catalysts, were also prepared. H_2PtCl_6/Fe_2O_3-600 and H_2PtCl_6-ethylene glycol/Fe_2O_3-600 catalysts were prepared by incipient wetness impregnation method with H_2PtCl_6 or H_2PtCl_6-ethylene glycol and homemade FeOOH support. Followed by same drying and RTT procedures, both catalysts were prepared. Pt_1/CeO_2-600 and Pt_1/AC-600 were also prepared by adsorption of Pt-en precursor on CeO_2 or AC support. And after same filtration, washing, drying and RTT procedures, both catalysts were prepared.

In the sample characterization section, the actual Pt loadings were determined by inductively coupled plasma spectroscopy (ICP-AES) on an IRIS Intrepid II XSP instrument (Thermo Electron Corporation).

X-ray diffraction (XRD) patterns were recorded on a PANalytical X' pert diffractometer with a Cu-Kα radiation source (40 kV and 40 mA). A continuous mode was used to record data in the 2θ range from 10° to 80°.

The thermogravimetric analysis (TGA) and differential scanning calorimeter (DSC) experiments of the catalysts was carried out in flowing air (100 mL min^{-1}) from 30 ^oC to 600 ^oC at a heating rate of 10 ^oC min^{-1} on the TA Instrument SDT Q600.

N_2 adsorption-desorption experiments were conducted on a Micromeritics ASAP-2010 physical adsorption apparatus. Before the measurement, the sample was pretreated at 110 ^oC for 12 h in vacuum. The specific surface area was calculated using a Brunauer−Emmett−Teller (BET) method.

Scanning transmission electron microscopy (STEM) and energy dispersive X-ray spectroscopy (EDS) experiments were performed on a JEOL JEM-2100F microscope operated at 200 kV, equipped with an Oxford Instruments ISIS/INCA energy-dispersive X-ray spectroscopy (EDS) system with an Oxford Pentafet Ultrathin Window (UTW) Detector. The aberration-corrected high-angle annual dark-filed scanning transmission electron microscopy (AC-HAADF-STEM) analysis was performed on a JEOL JEM-ARM200F equipped with a CEOS probe corrector, with a guaranteed resolution of 0.08 nm. Before microscopy examination, the sample was ultrasonically dispersed in ethanol for 15-20 min, and then a drop of the suspension was dropped on a copper TEM grid coated with a thin holey carbon film.

The X-ray absorption spectra (XAS) including X-ray absorption near edge structure (XANES) and extended X-ray absorption fine structure (EXAFS) at Pt L_{III}-edge of the samples were measured

at the beamline 14W of Shanghai Synchrotron Radiation Facility (SSRF) in China. The output beam was selected by Si(111) monochromator, and the energy was calibrated by Pt foil. Before measurement, the samples were diluted by boron nitride and tableted. The data were collected at room temperature under fluorescence mode by using Lytle detector. Athena software package was employed to process the XAS data.

In the XAFS results, the contribution of Pt-C arises from the $(Pt(en)_2)^{2+}$ complex, as shown in Figure 3d The C species is located on the Pt second shell. After adsorption of the $(Pt(en)_2)^{2+}$ complex on the FeOOH support, the Pt-C contribution still existed, but it was absent from the samples after the RTT treatment at high temperatures owing to the decomposition of the en ligand. Therefore, in the EXAFS spectra (Figure 3a), only the two samples, Pt-en (liquid) and the Pt_1/FeOOH-RT, present the Pt-C contributions at around 2.5 Å, which coincidently overlaps with the Pt-Pt contribution. But there aren't any Pt-Pt contributions in both samples

X-ray photoelectron spectroscopy (XPS) spectra were obtained on a Thermo ESCALAB 250 X-ray photoelectron spectrometer equipped with Al Kα excitation source and with C as internal standard (C 1*s* = 284.6 eV).

H_2-microcalorimetric measurement was performed by a BT2.15 heat-flux calorimeter, which was connected to a gas handling and a volumetric system employing MKS Baratron Capacitance Manometers for precision pressure measurement. The ultimate dynamic vacuum of the microcalorimetric system was 10^{-7} Torr by calculation. First, the fresh sample was treated in a special cell in high pure He at 120 ^{o}C to eliminate the adsorption. Then, the sample was transferred and sealed in a Pyrex capsule without exposure to Air. After that, the capsule was placed into the the high vacuum system and stabilized for (6-8 h). After thermal equilibrium was reached, the capsule was broken by a vacuum feedthrough. And the H_2-microcalorimetric data was collected by sequentially introducing small doses (10^{-6} mol) of H_2 into the system until it became saturated (5-6 Torr). Simultaneously, the differential heat versus adsorbate coverage plots and adsorption isothermals can be obtained after a typical H_2-microcalorimetric experiment.

In the computational details section, all the calculations were performed using periodic DFT methods as implemented in the Vienna ab-initio simulation package (VASP).[1,2] Projector augmented wave (PAW) method was used for the interaction between the atomic cores and valence electrons.[3] The valence orbitals of Fe (3*d*, 4*s*), O (2*s*, 2*p*) and H (1*s*) were described by plane-wave basis sets

with cutoff energies of 400 eV. The exchange-correlation energies were calculated via the generalized gradient approximation (GGA) with the PBE functional.[4] Gaussian smearing method with a width of 0.05 eV was used. Spin-polarized DFT+U calculations [5,6] with a value of U_{eff} = 3.0 eV [7,8] was used to describe the localized Fe 3*d* states. The Brillouin zone was sampled at the Γ-point. The convergence criteria for the energy and force were set to 10^{-5} eV and 0.02 eV $Å^{-1}$. The transition state (TS) of the surface reaction was searched using the dimer method.[9] Vibrational analysis was further used to confirm the transition states with only one imaginary frequency. The energy barrier (Ea) was determined as the energy difference between the corresponding transition- and initial-states. The *adsorption energies* was calculated according to the supplementary equation (1).

$$E_{\text{ads}} = E_{(\text{slab + adsorbate})} - E_{(\text{slab})} - E_{(\text{adsorbate})} \qquad \text{Supplementary Equation (1)}$$

In supplementary equation (1), $E_{(\text{slab + adsorbate})}$, $E_{(\text{slab})}$, and $E_{(\text{adsorbate})}$ are the energies of species adsorbed on the surface, the bare surface, and the gas-phase molecule, respectively. The *reaction energy* was calculated by supplementary equation (2).

$$\Delta E = E_{(\text{products})} - E_{(\text{reactants})} \qquad \text{Supplementary Equation (2)}$$

Atomic charges were computed using the atom-in-molecule (AIM) scheme proposed by Bader.[10]

The α-Fe_2O_3-*p*(4 × 4) (0001) surface was modeled by *p(*4 × 4) supercells containing 12 layers of Fe atoms and 7 atomic layers of O_3 to model the O-terminated surface. The 10 top-layer slabs of the surface were allowed to relax while the other layers beneath the surface were frozen during the geometry optimizations. The vacuum gap was set as ~15 Å to avoid the interaction between periodic images. All supercell slabs were periodically repeated with a 15 Å vacuum layer between surfaces in the direction of the surface normal. Antiferromagnetic properties of α-Fe_2O_3 were represented by a (+ – – +) magnetic configuration, which was proven to be the most energetically favorable for α-Fe_2O_3 previously.[11]

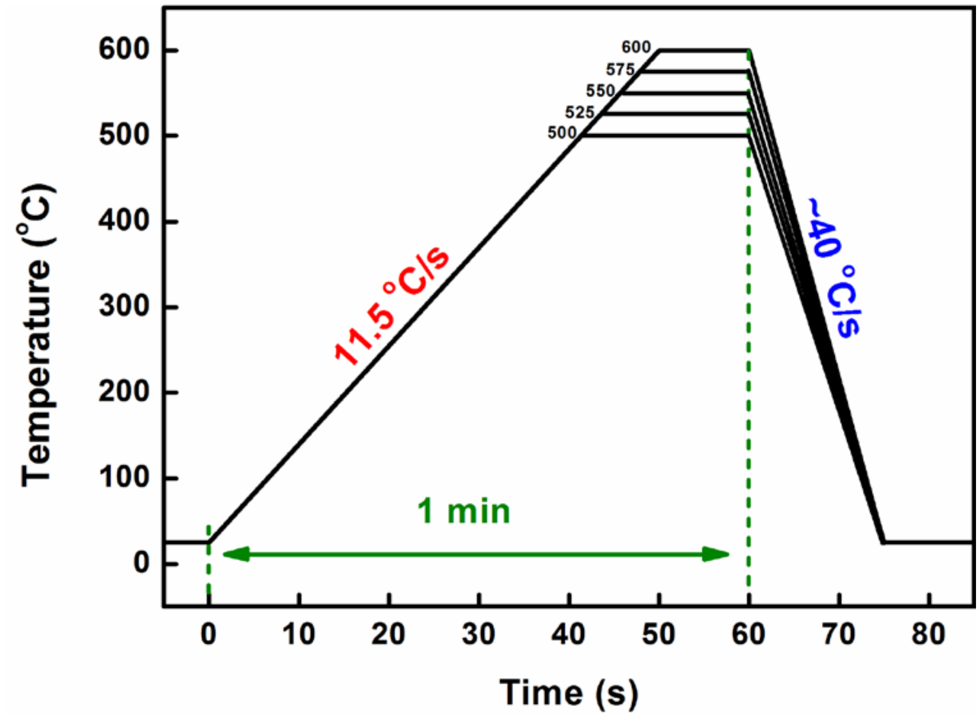

Supplementary Figure 1. The temperature profile of the sample during the RTT process.

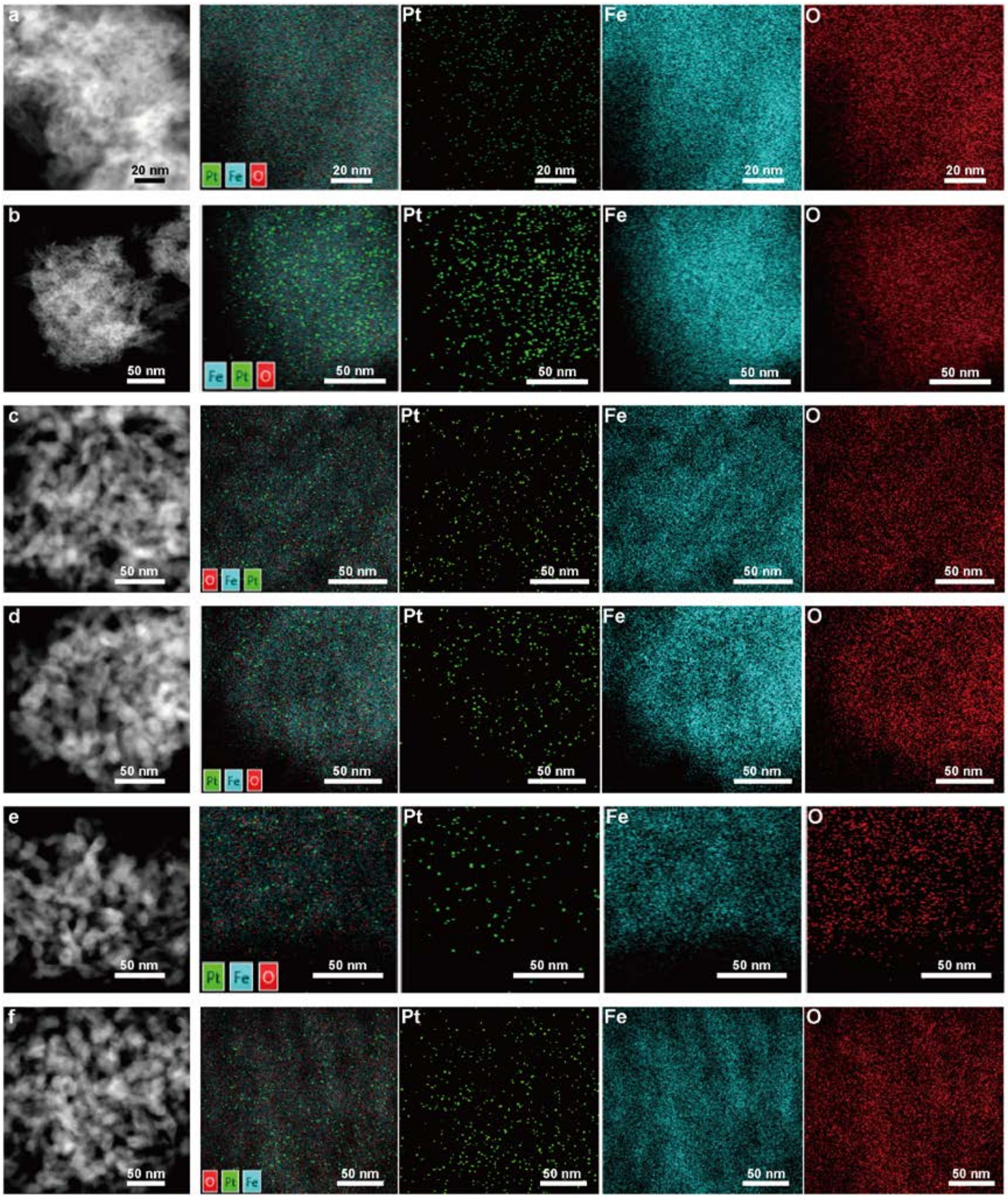

Supplementary Figure 2. HAADF-STEM and corresponding element mapping images of Pt_1/Fe_2O_3-T catalysts at low magnification. The absence of any nanoparticles in the images as well as the uniform Pt signals in the mapping suggest that Pt species are highly dispersed on the Pt_1/FeOOH-RT **(a)**, Pt_1/Fe_2O_3-500 **(b)**, Pt_1/Fe_2O_3-525 **(c)**, Pt_1/Fe_2O_3-550 **(d)**, Pt_1/Fe_2O_3-575 **(e)**, and Pt_1/Fe_2O_3-600 **(f)** catalysts.

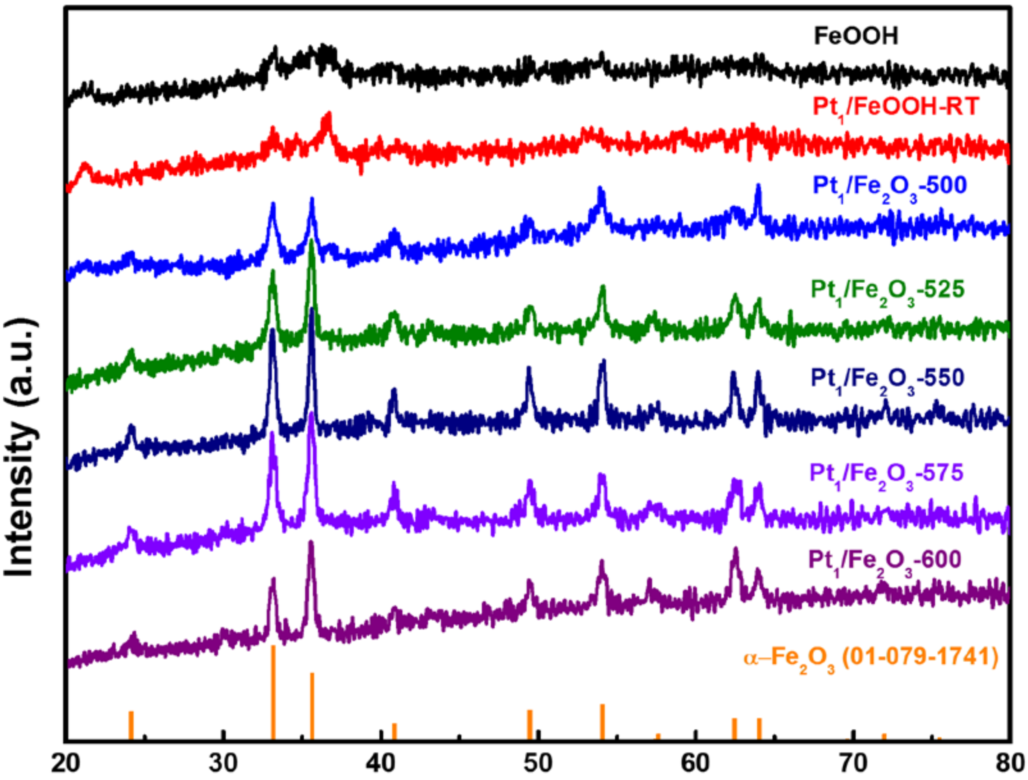

Supplementary Figure 3. The XRD patterns of the series of Pt_1/Fe_2O_3-T catalysts as well as that of FeOOH support. The absence of reflections of Pt or Pt oxide indicates that all the Pt species are highly dispersed.

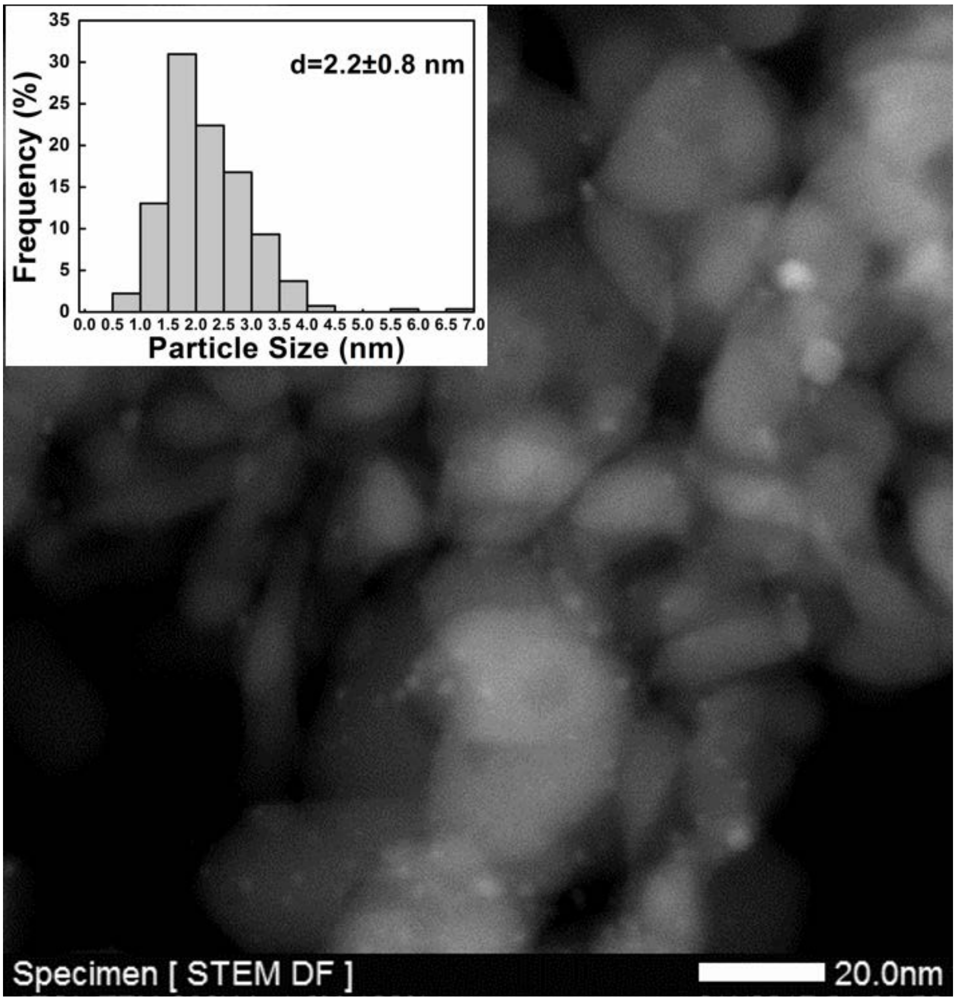

Supplementary Figure 4. HAADF-STEM image of H_2PtCl_6/Fe_2O_3-600 catalyst without use of the en ligand. Pt nanoparticles are clearly observed in the image. The inset is the histogram of the Pt particle size distribution.

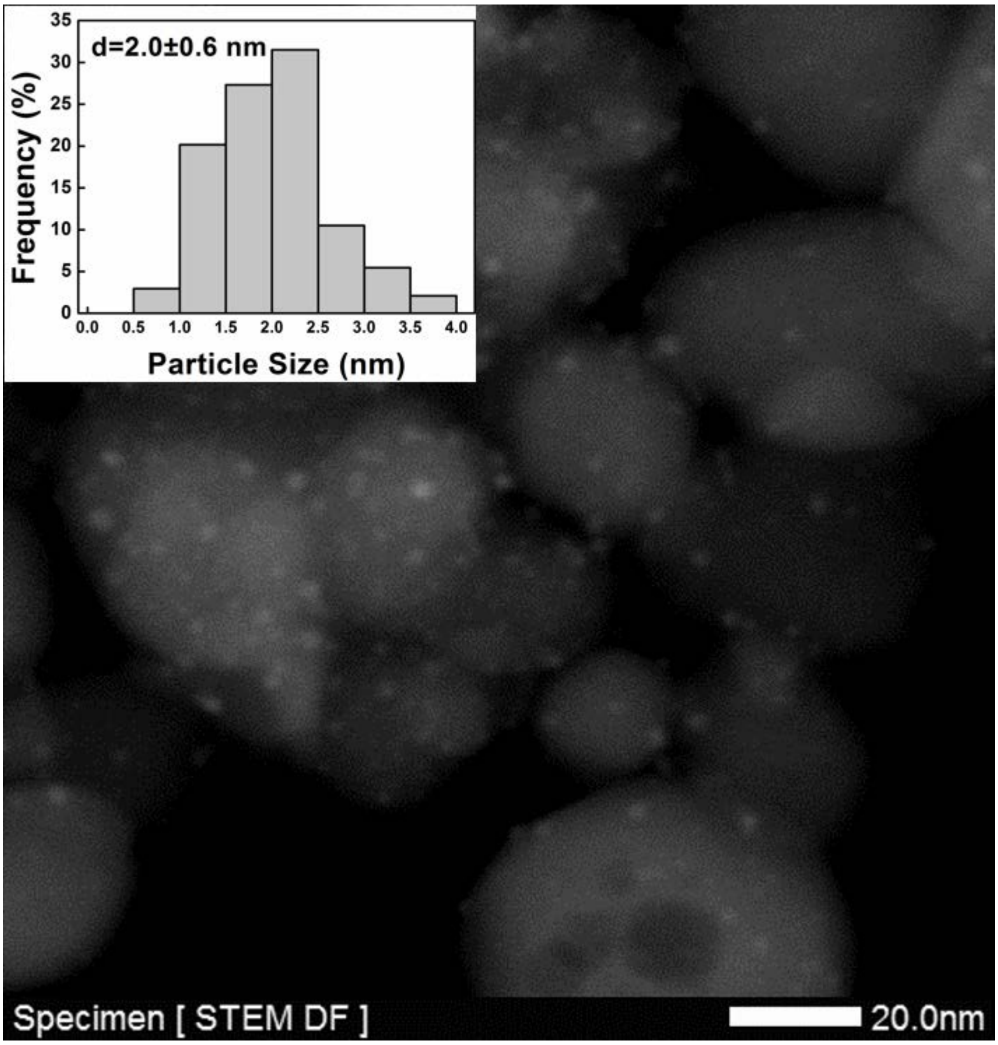

Supplementary Figure 5. HAADF-STEM image of H_2PtCl_6-ethylene glycol/Fe_2O_3-600 catalyst. The catalyst was prepared with ethylene glycol to replace en ligand. Pt nanoparticles are clearly observed in the image. The inset is the histogram of the Pt particle size distribution.

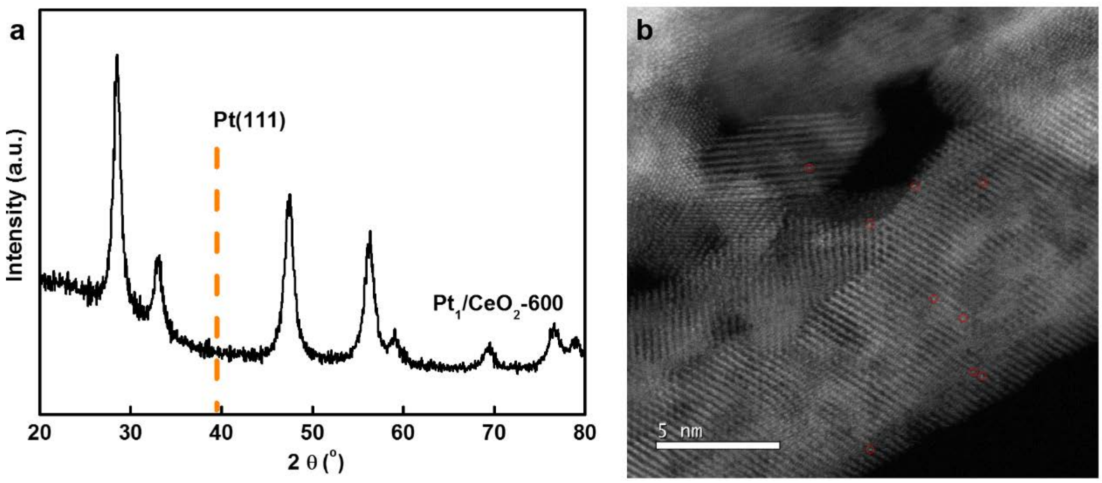

Supplementary Figure 6. XRD pattern (a) and AC-HAADF-STEM image (b) of Pt_1/CeO_2-600 catalyst. The absence of Pt reflection peaks in the XRD pattern and the absence of Pt aggregates in the HAADF-STEM images indicates the single-atom dispersion of Pt on the CeO_2 support.

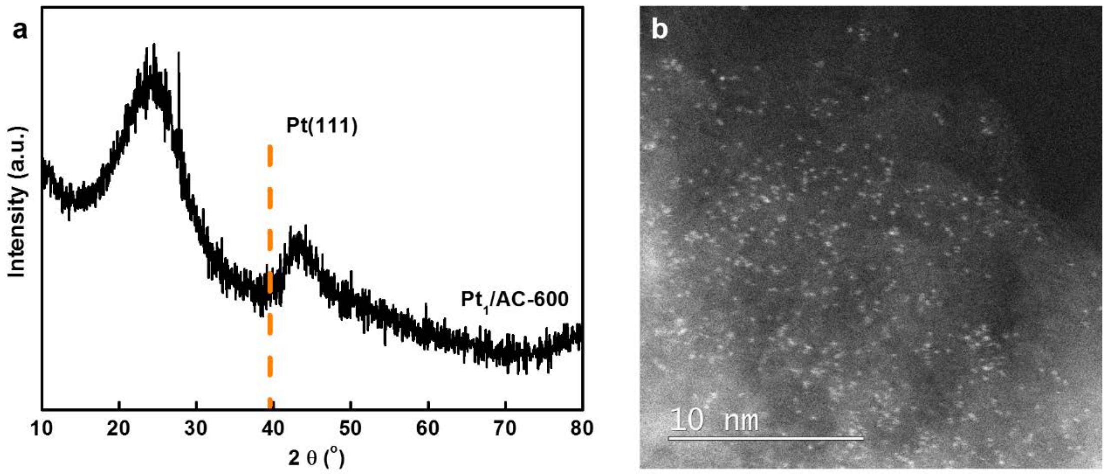

Supplementary Figure 7. XRD pattern (a) and AC-HAADF-STEM image (b) of Pt_1/AC-600 catalyst. The absence of Pt reflection peaks in the XRD pattern and the absence of Pt aggregates in the HAADF-STEM images indicates the single-atom dispersion of Pt on the AC support.

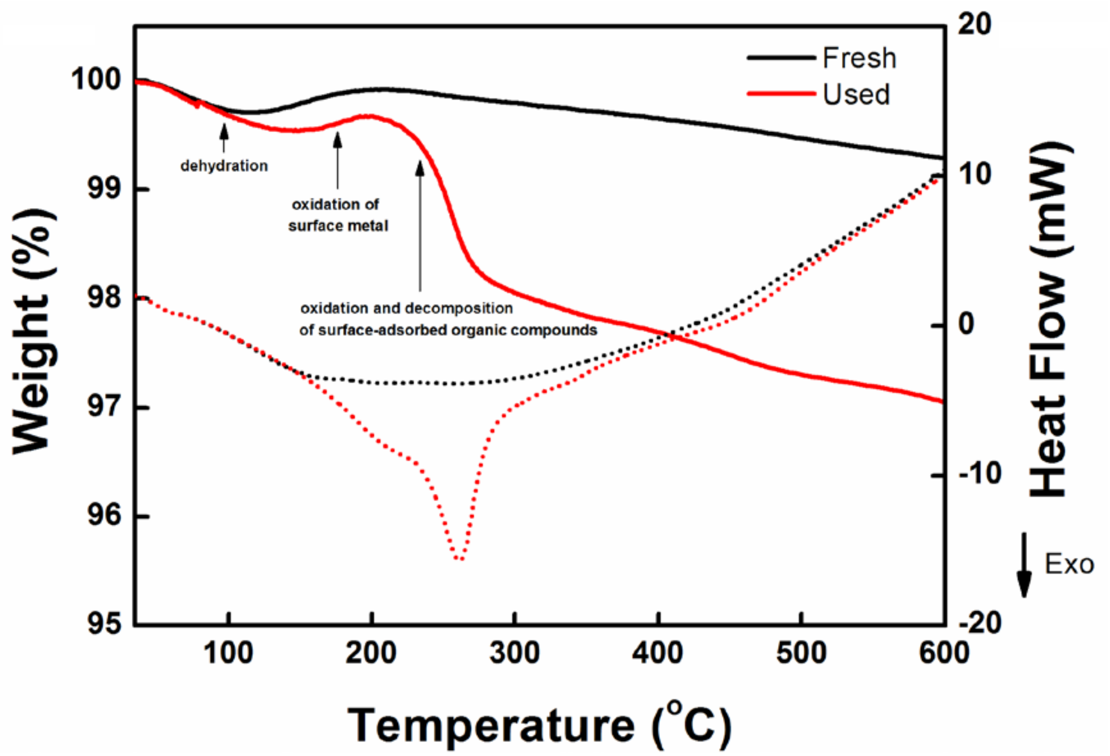

Supplementary Figure 8. TGA/DSC profile of the Pt_1/Fe_2O_3-600-used sample. There is a weight loss of 1.5% at around 250 °C, indicating there is organic residue covering the active sites, which led to the slight decay of activity. (TGA: solid line; DSC: dotted line)

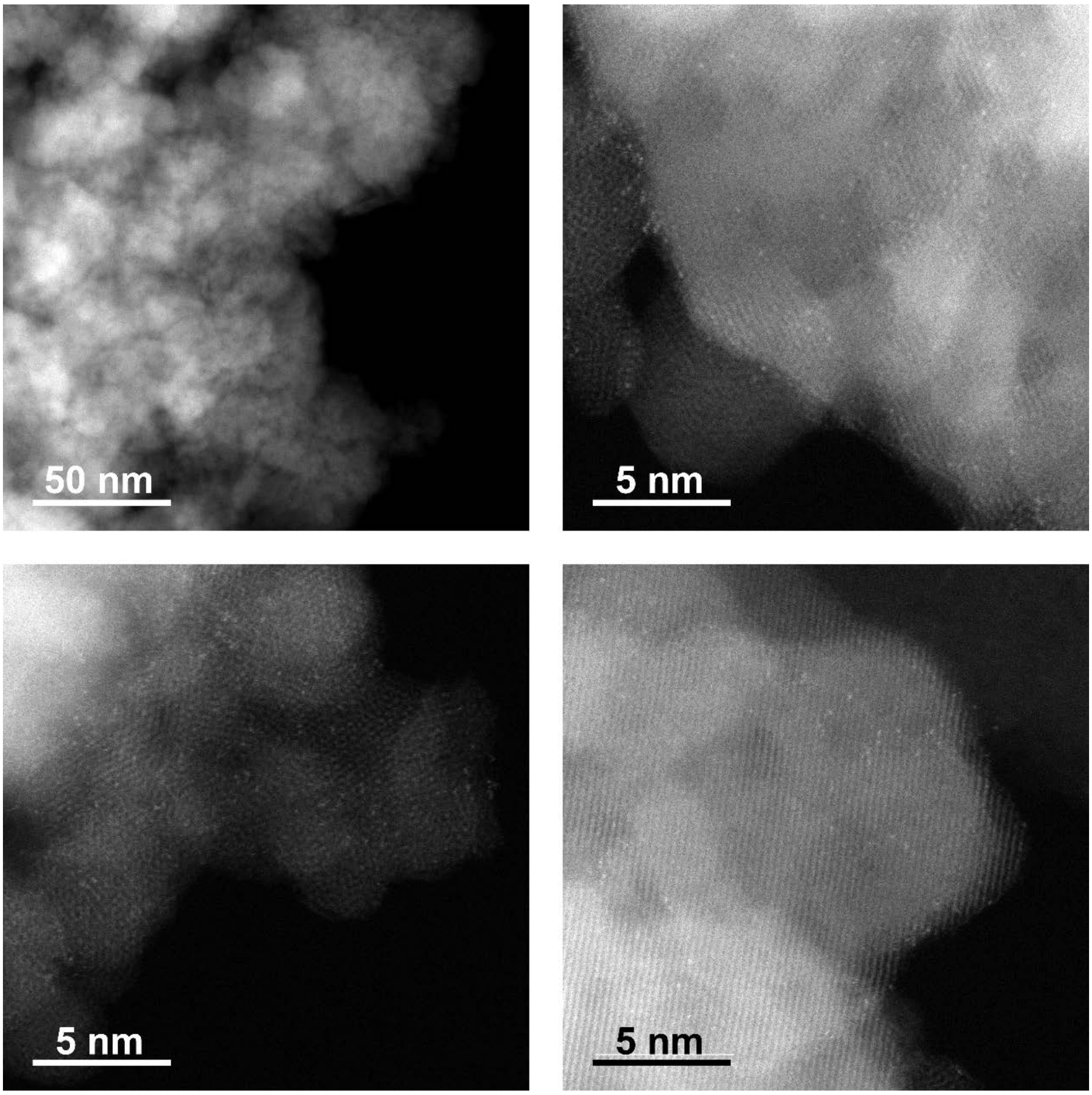

Supplementary Figure 9. AC-HAADF-STEM images of Pt_1/Fe_2O_3-600-used catalyst. All the Pt species are atomically dispersed without any aggregation.

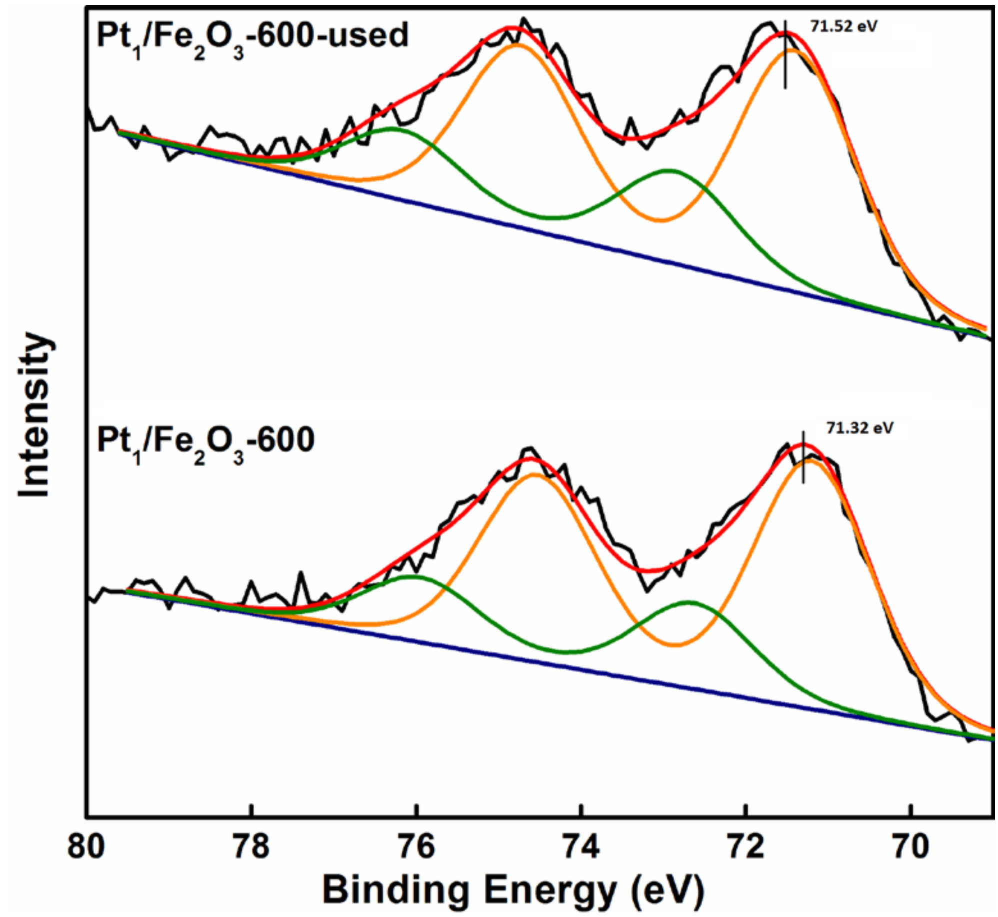

Supplementary Figure 10. Pt 4*f* XPS spectra of Pt_1/Fe_2O_3-600 and Pt_1/Fe_2O_3-600-used samples. The Pt 4*f* binding energy remain essentially unchanged after the reaction, demonstrating that the chemical state of Pt single atoms did not change after the reaction.

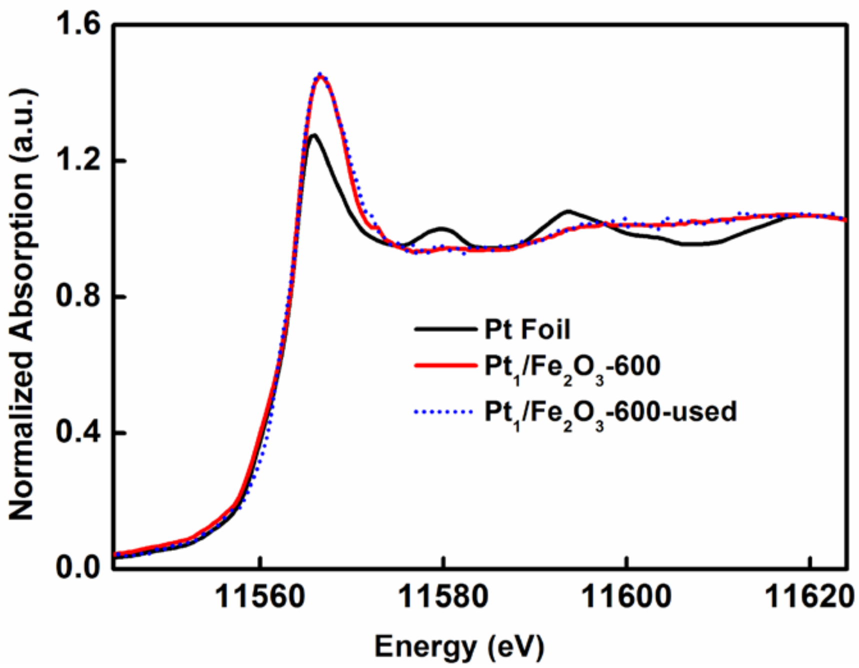

Supplementary Figure 11. The normalized XANES spectra at the Pt L_{III} edge of Pt_1/Fe_2O_3-600 and Pt_1/Fe_2O_3-600-used catalysts. The Pt XANES spectra almost unchanged after the reaction, demonstrating that the chemical environment (Pt-O coordination number and oxidation state) of Pt single atoms did not change after the reaction.

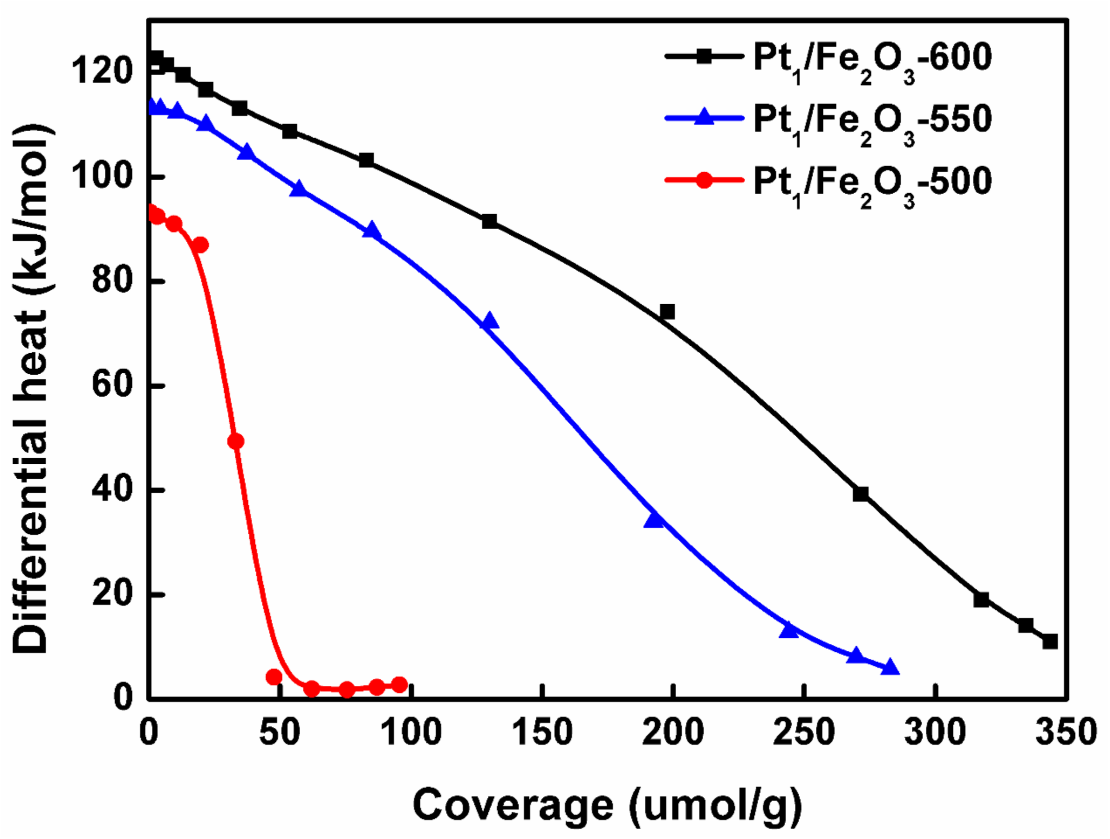

Supplementary Figure 12. Differential adsorption heat of hydrogen as a function of coverage on the Pt_1/Fe_2O_3-T catalysts. Adsorption was conducted at 40 °C.

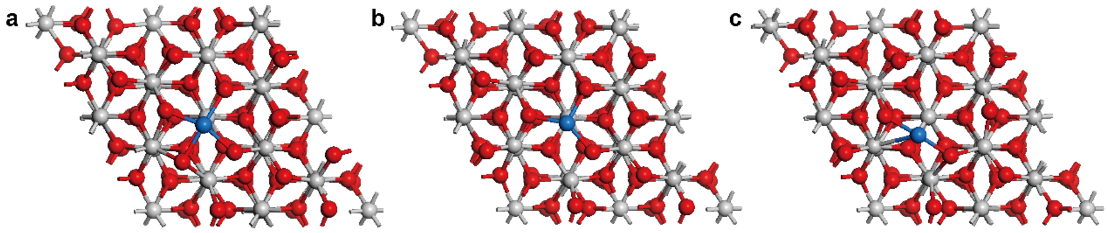

Supplementary Figure 13. The optimized structures of the Pt_1/Fe_2O_3-T catalysts. (a) four-coordinated Pt_1/Fe_2O_3-500; (b) three-coordinated Pt_1/Fe_2O_3-550; (c) two-coordinated Pt_1/Fe_2O_3-600. Color codes: grey (Fe), red (O) and blue (Pt). Based on the DFT calculation, the distances of Pt-O bonds are 1.90 Å, 1.93 Å, 1.95 Å and 1.99 Å for four-coordinated Pt_1/Fe_2O_3-500, 1.86 Å, 1.92 Å and 1.93 Å for three-coordinated Pt_1/Fe_2O_3-550 and 1.86 Å and 1.88 Å for two-coordinated Pt_1/Fe_2O_3-600. The Bader charge of single Pt atom is +1.48 |e| for four-coordinated Pt_1/Fe_2O_3-500, +1.31 |e| for three-coordinated Pt_1/Fe_2O_3-550 and +0.78 |e| for two-coordinated Pt_1/Fe_2O_3-600, respectively.

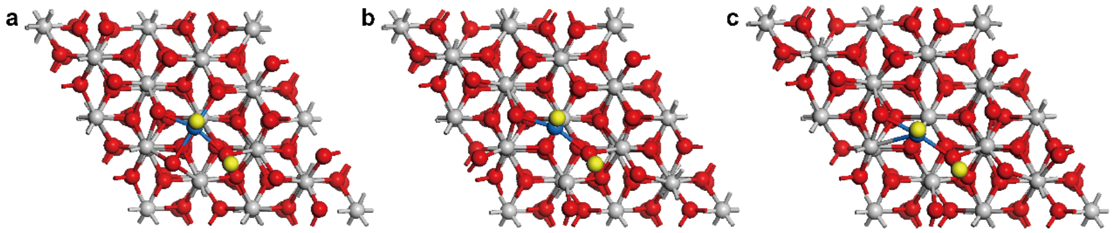

Supplementary Figure 14. The optimized structures for hydrogen adsorption on the Pt_1/Fe_2O_3-T catalysts. (a) four-coordinated Pt_1/Fe_2O_3-500; (b) three-coordinated Pt_1/Fe_2O_3-550; (c) two-coordinated Pt_1/Fe_2O_3-600. Color codes: grey (Fe), red (O), yellow (H) and blue (Pt). The adsorption energies of H_2 are -1.80 eV, -2.03 eV, and -2,19 eV on the four-coordinated Pt_1/Fe_2O_3-500, three-coordinated Pt_1/Fe_2O_3-550, and two-coordinated Pt_1/Fe_2O_3-600, respectively.

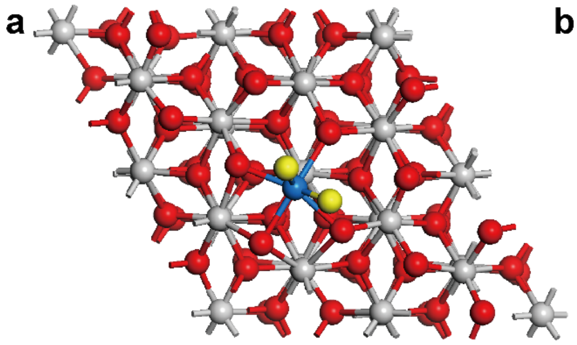

Supplementary Figure 15. The optimized structures of transition states for hydrogen adsorption on the Pt_1/Fe_2O_3-T catalysts. (a) four-coordinated Pt_1/Fe_2O_3-500; (b) three-coordinated Pt_1/Fe_2O_3-550. Color codes: grey (Fe), red (O), yellow (H) and blue (Pt).

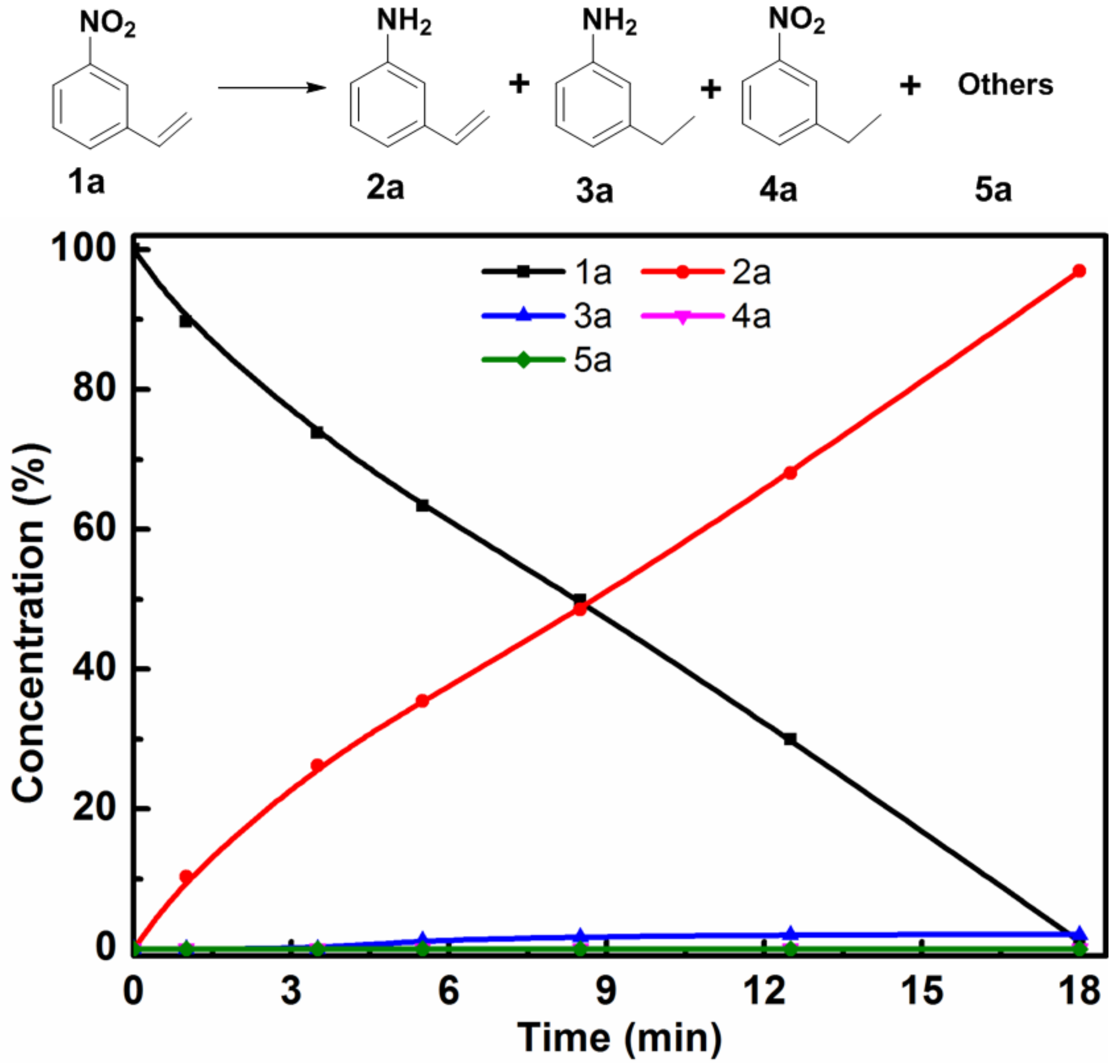

Supplementary Figure 16. The concentration change of reactant, product and by-products during the chemoselective hydrogenation of 3-nitrostyrene over Pt_1/Fe_2O_3-600 catalyst.

Supplementary Table 1. The Pt loadings, average oxidation state of Pt and surface Pt/Fe atomic ratios of the Pt_1/Fe_2O_3-T catalysts.

Sample	Pt loading (wt%)[a]	Binding energy of Pt $4f_{7/2}$	Content (%)[b]	Average oxidation state of Pt from XPS (XANES) results[c]	Surface Pt/Fe atomic ratio (%)[d]
Pt_1/FeOOH -RT	0.8	75.18 (Pt^{IV})<br>72.78 (Pt^{II})	24.0<br>76.0	2.48 (2.46)	1.00
Pt_1/Fe_2O_3 -500	0.9	72.76 (Pt^{II})<br>71.30 (Pt^0)	87.0<br>13.0	1.74 (1.81)	1.00
Pt_1/Fe_2O_3 -525	0.9	72.30 (Pt^{II})<br>71.20 (Pt^0)	62.7<br>37.3	1.25 (1.36)	1.51
Pt_1/Fe_2O_3 -550	0.9	72.30 (Pt^{II})<br>71.20 (Pt^0)	51.8<br>48.2	1.04 (1.23)	1.42
Pt_1/Fe_2O_3 -575	0.9	72.48 (Pt^{II})<br>71.30 (Pt^0)	32.1<br>67.9	0.64 (0.66)	1.64
Pt_1/Fe_2O_3 -600	0.9	72.62 (Pt^{II})<br>71.20 (Pt^0)	25.7<br>74.3	0.51 (0.56)	1.92

[a] The Pt loading was determined by ICP-AES.

[b] The Pt species content error is approximately 3%.[12]

[c] For XPS analysis, the average oxidation state of Pt is calculated by supplementary equation (3):

$$Pt_{\text{Average oxidation state}} = \text{Content }\%_{(Pt(IV))} * 4 + \text{Content }\%_{(Pt(II))} * 2 + \text{Content }\%_{(Pt(0))} * 0$$

Supplementary Equation (3)

For XANES analysis, the average oxidation state of Pt is determined by the solution described by Lytle et al.[13] and Horsley.[14] Firstly, the Pt L_{III} XANES spectra peak area of the Pt foil, PtO_2 references and a series of Pt_1/Fe_2O_3-T catalysts are fitted by setting the erf function as baseline and setting the Gaussian function as fitted curve. Then the sum of the erf function and Gaussian function with the XANES spectrum is fitted. Finally, due to the linear relationship of the white line area and

the center Pt atom oxidation state[15-17] and by correlating the Gaussian function area of the references and the catalysts, average oxidation state of Pt in catalyst is determined. The XANES area error is approximately 5%.[14,18]

[d] The surface Pt/Fe atomic ratio was calculated according to supplementary equation (4):

$$\frac{n_i}{n_j} = \frac{I_i}{I_j} \times \frac{\sigma_j}{\sigma_i} \times \frac{E_{k_j}^{0.5}}{E_{k_i}^{0.5}} \qquad \text{Supplementary Equation (4)}$$

i: Fe species; j: Pt species; n: the numbers of surface atom; I: the intensity of XPS peak area; σ: photoionization cross section; $Al_{K\alpha}$: Fe 2$p_{3/2}$ = 10.82, Pt 4$f_{7/2}$ = 8.65; E_k: photoelectron kinetic energy.

E_k = hν ($Al_{K\alpha}$, hν = 1486.6 eV) – BE (Binding Energy) Supplementary Equation (5)

And the theoretical value of the Pt/Fe atomic ratio was calculated according to the loading of Pt in Pt_1/Fe_2O_3-T catalyst.

Supplementary Table 2. Performance comparison of Pt_1/Fe_2O_3-600 catalyst with other catalysts reported in literature for the hydrogenation of 3-nitrostyrene to 3-vinylaniline.

Cat.	Temp. (°C)	P (bar)	Time (min)	Conv. (%)	Sel. (%)	TOF ($mol_{conv.}h^{-1}mol_{Pt}^{-1}$)	Ref.
Pt_1/Fe_2O_3-600	40	3	18	98.9	98.0	3809	This work
Pt_1/Fe_2O_3-600	60	10	6	97.2	96.2	21099	This work
Pt/FeOx	40	3	50	96.5	98.6	1514	20
Na-Pt/FeOx	40	3	32	95.1	97.5	1083	19
Pt/TiO_2	40	3	390	95.1	93.1	60	21
Pt/ZnO	75	10	-	-	97	12.4	22
Au/TiO_2	120	8	360	98.5	95.9	173	23
$Ag@CeO_2$	110	6	360	99	99	4.85	24
$RhIn/SiO_2$	75	1	120	99	91	26.1	25
$Au/Sn\text{-}TiO_2$	70	13	240	99	99.3	319	26
$Pt/Sn\text{-}TiO_2$	45	2	120	98.5	97.4	1802	26
Au/ZnAl-HT	90	10	200	100	>99	76	27
$PtNi/SiO_2$	40	3	100	>99	>99	1800	28

Supplementary Table 3. The reusability of Pt_1/Fe_2O_3-600 catalyst for the chemoselective hydrogenation of 3-nitrostyrene.

Run number	Time (min)	Conv. (%)	Sel. (%)
1	18	98.9	98.0
2	18	98.9	99.0
3	19	95.5	97.1
4	22	95.5	95.1
5	25	96.3	98.1

Pre-treatment condition: 5ml toluene, 10 bar H_2, 40 °C, 1h. Reaction conditions: 40 °C, 3 bar H_2, 10 mg catalyst and 90 mg Fe_2O_3. 5ml reaction mixture, 0.5 mmol substrate, toluene as solvent, o-xylene as internal standard.

Supplementary Table 4. ICP and XPS analysis results of the Pt_1/Fe_2O_3-600 and Pt_1/Fe_2O_3-600-used catalysts

Sample	Pt loading determined by ICP (%)	Pt/Fe surface atomic ratio determined by XPS (%)	Binding energy of Pt 4$f_{7/2}$
Pt_1/Fe_2O_3-600	0.9	1.92	71.32
Pt_1/Fe_2O_3-600-used	0.9	2.05	71.52

Supplementary Table 5. Chemoselective hydrogenation of different substituted nitroarenes to the corresponding anilines over the Pt_1/Fe_2O_3-600 catalyst.

Entry	Substrate	Product	Time (min)	Yield (%)
1	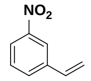	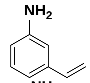	18	96.9
2	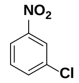	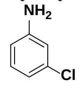	20	96.4
3	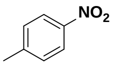	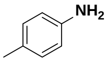	18	96.5
4	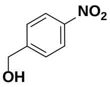	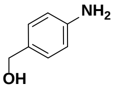	15	95.5
5	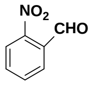	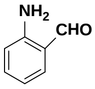	42	95.9
6	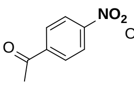	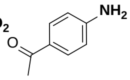	22	94.8
7*	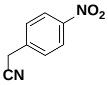	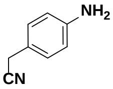	180	97.2
8*	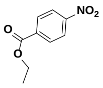	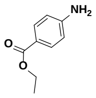	120	98.1

Pre-treatment condition: 5ml toluene, 10 bar H_2, 40 ^{o}C, 1h. Reaction conditions: 40 ^{o}C, 3 bar H_2, 10 mg catalyst and 90 mg Fe_2O_3. 5ml reaction mixture, 0.5 mmol substrate, toluene as solvent. Isolated yield. * 50 ^{o}C, 6 bar H_2, 20 mg catalyst and 80 mg Fe_2O_3.

Supplementary Table 6. H_2 uptake and H_2/Pt ratio of Pt_1/Fe_2O_3-T catalysts at 40 kJ mol^{-1} based on the microcalorimetry experiment.

Catalyst	H_2 uptake at 40 kJ mol^{-1} (umol g^{-1})	H_2/Pt ratio
Pt_1/Fe_2O_3-500	36.1	0.78
Pt_1/Fe_2O_3-550	183.8	3.98
Pt_1/Fe_2O_3-600	270.4	5.86

Supplementary Table 7. Kinetic isotope effect experiment of chemoselective hydrogenation of 3-nitrostyrene over Pt_1/Fe_2O_3-500 and Pt_1/Fe_2O_3-600 catalysts. [a]

Catalyst	$TOF_{hydrogen}$ ($mol_{conv.} h^{-1} mol_{Pt}^{-1}$)	$TOF_{deuterium\ gas}$ ($mol_{conv.} h^{-1} mol_{Pt}^{-1}$)	KIE (r H_2/r D_2)
Pt_1/Fe_2O_3-500	491	75	6.55
Pt_1/Fe_2O_3-600	3809	1873	2.03

Pre-treatment condition: 5ml toluene, 10 bar, 40 ^oC, 1h. Reaction conditions: 40 ^oC, 3 bar, 10 mg catalyst and 90 mg Fe_2O_3. 5ml reaction mixture, 0.5 mmol substrate, toluene as solvent, o-xylene as internal standard.

[a] The KIE value was calculated using supplementary equation (6):

$$KIE = (TOF_{hydrogen})/(TOF_{deuterium\ gas}) \quad \text{Supplementary Equation (6)}$$

Supplementary Reference

1. Kresse, G. & Furthmüller, J. Efficiency of ab-initio total energy calculations for metals and semiconductors using a plane-wave basis set. *Comput. Mater. Sci.* **6,** 15-50 (1996).
2. Kresse, G. & Furthmüller, J. Efficient iterative schemes for ab initio total-energy calculations using a plane-wave basis set. *Phys. Rev. B* **54,** 11169-11186 (1996).
3. Kresse, G. & Joubert, D. From ultrasoft pseudopotentials to the projector augmented-wave method. *Phys. Rev. B* **59,** 1758-1775 (1999).
4. Perdew, J. P., Burke, K. & Ernzerhof, M. Generalized gradient approximation made simple. *Phys. Rev. Lett.* **77,** 3865-3868 (1996).
5. Dudarev, S. L., Botton, G. A., Savrasov, S. Y., Humphreys, C. J. & Sutton, A. P. Electron-energy-loss spectra and the structural stability of nickel oxide: An LSDA+U study. *Phys. Rev. B* **57,** 1505-1509 (1998).
6. Anisimov, V. I., Aryasetiawan, F. & Lichtenstein, A. First-principles calculations of the electronic structure and spectra of strongly correlated systems: the LDA+U method. *J. Phys.: Condens. Matter.* **9,** 767-708 (1997).
7. Qiao, B. et al. Single-atom catalysis of CO oxidation using Pt_1/FeO_x. *Nat. Chem.* **3,** 634-641 (2011).
8. Rollmann, G., Rohrbach, A., Entel, P. & Hafner, J. First-principles calculation of the structure and magnetic phases of hematite. *Phys. Rev. B* **69,** 165107 (2004).
9. Henkelman, G. & Jónsson, H. A dimer method for finding saddle points on high dimensional potential surfaces using only first derivatives. *J. Chem. Phys.* **111,** 7010-7022 (1999).
10. Bader, R. F. A quantum theory of molecular structure and its applications. *Chem. Rev.* **91,** 893-928 (1991).
11. Sandratskii, L. M., Uhl, M. & Kübler, J. Band theory for electronic and magnetic properties of α-Fe_2O_3. *J. Phys.: Condens. Matter.* **8,** 983-989 (1996).
12. Aronniemi, M., Sainio, J. & Lahtinen, J. Chemical state quantification of iron and chromium oxides using XPS: the effect of the background subtraction method. *Surf. Sci.* **578,** 108-123 (2005).
13. Lytle, F. W., Wei, P. S. P., Greegor, R. B., Via, G. H. & Sinfelt, J. H. Effect of chemical

environment on magnitude of x-ray absorption resonance at L_{III} edges. Studies on metallic elements, compounds, and catalysts. *J. Chem. Phys.* **70,** 4849-4855 (1979).

14. Horsley, J. A. Relationship between the area of $L_{2,3}$ x-ray absorption edge resonances and the d orbital occupancy in compounds of platinum and iridium. *J. Chem. Phys.* **76,** 1451-1458 (1982).
15. Cui, Y. T. et al. Wetting Induced Oxidation of Pt-based Nano Catalysts Revealed by In Situ High Energy Resolution X-ray Absorption Spectroscopy. *Sci. Rep.* **7,** 1482 (2017).
16. Ota, N., Tamura, M., Nakagawa, Y., Okumura, K. & Tomishige, K. Performance, Structure, and Mechanism of ReO_x−Pd/CeO_2 Catalyst for Simultaneous Removal of Vicinal OH Groups with H_2. *ACS Catal.* **6,** 3213-3226 (2016).
17. Nakagawa, Y. et al. Mechanistic Study of Hydrogen-Driven Deoxydehydration over Ceria-Supported Rhenium Catalyst Promoted by Au Nanoparticles. *ACS Catal.* **8,** 584-595 (2018).
18. Hall, M. D., Foran, G. J., Zhang, M., Beale, P. J. & Hambley, T. W. XANES determination of the platinum oxidation state distribution in cancer cells treated with platinum (IV) anticancer agents. *J. Am. Chem. Soc.* **125,** 7524-7525 (2003).
19. Wei, H. et al. FeO_x-supported platinum single-atom and pseudo-single-atom catalysts for chemoselective hydrogenation of functionalized nitroarenes. *Nat. Commun.* **5,** 5634 (2014).
20. Wei, H. et al. Remarkable effect of alkalis on the chemoselective hydrogenation of functionalized nitroarenes over high-loading Pt/FeO_x catalysts. *Chem. Sci.* **8,** 5126-5131 (2017).
21. Corma, A., Serna, P., Concepción, P. & Calvino, J. J. Transforming nonselective into chemoselective metal catalysts for the hydrogenation of substituted nitroaromatics. *J. Am. Chem. Soc.* **130,** 8748-8753 (2008).
22. Berguerand, C. et al. Chemoselective Liquid Phase Hydrogenation of 3-Nitrostyrene over Pt Nanoparticles: Synergy with ZnO Support. *Ind. Eng. Chem. Res.* **54,** 8659-8669 (2015).
23. Corma, A. & Serna, P. Chemoselective hydrogenation of nitro compounds with supported gold catalysts. *Science* **313,** 332-334 (2006).
24. Mitsudome, T. et al. Design of a silver-cerium dioxide core-shell nanocomposite catalyst for chemoselective reduction reactions. *Angew. Chem. Int. Ed.* **51,** 136-139 (2012).
25. Furukawa, S., Takahashi, K. & Komatsu, T. Well-structured bimetallic surface capable of

molecular recognition for chemoselective nitroarene hydrogenation. *Chem. Sci.* **7,** 4476-4484 (2016).

26. Wang, L. et al. Single-site catalyst promoters accelerate metal-catalyzed nitroarene hydrogenation. *Nat. Commun.* **9,** 1362 (2018).
27. Tan, Y. et al. ZnAl-Hydrotalcite-Supported Au_{25} Nanoclusters as Precatalysts for Chemoselective Hydrogenation of 3-Nitrostyrene. *Angew. Chem. Int. Ed.* **56,** 2709-2713 (2017).
28. Peng, Y. et al. Pt Single Atoms Embedded in the Surface of Ni Nanocrystals as Highly Active Catalysts for Selective Hydrogenation of Nitro Compounds. *Nano Lett.* **18,** 3785-3791 (2018).
