# Supplementary material for: Unraveling the coordination structure-performance relationship in Pt1/Fe2O3 single-atom catalyst
Source: Nat Commun. 2019 Oct 3;10:4500. doi: 10.1038/s41467-019-12459-0 (PMC6776505; doi:10.1038/s41467-019-12459-0)
Supplement: Supplementary file 2 — Peer Review [file 41467_2019_12459_MOESM2_ESM.pdf]

Reviewers' comments:

Reviewer #1 (Remarks to the Author):

Authors report a novel method to prepare Pt single-atom catalysts with tunable coordination sphere aiming at establishing structure-performance relationship for the chemoselective hydrogenation of 3-nitrostyrene. A detailed characterization of active species with distinct Pt-O coordination numbers is provided with clear conclusions. The Reviewer thinks that this work is of high relevance and has the potential to be published in Nat Commun. However, many clarifications and additional experiments are needed to meet the high standards required in this authoritative journal. The points to be addressed before publications are the following:
1. After the preparation of the catalysts, how can authors be sure that the chlorine complex is removed, but not deposited on the catalyst? Even if the reaction between chloroplatinic acid and ethanediamine yields 100% (which the Reviewer assumes not true), the residual chlorine stays in the solution and potentially may be deposited on the Fe2O3 surface. Then, the peak around 2.5 Å shown in the EXAFS spectra (Fig. 3a) could correspond to Pt-Cl bond and gradually decrease with elevating the treatment temperature (please refer to: https://doi.org/10.1016/S0920-5861(98)00267-3; doi:10.1006/jcat.2002.3601). Therefore, the presence of chlorine can act as a poison for Pt1/Fe2O3-500, -525 materials.
2. 1 min of keeping the sample in the furnace is not enough to reach thermal equilibrium due to the heat transfer and, thus, the samples actually were not heated up to reported temperatures- evidences are required in order to state that the set temperature was reached by the sample.
3. Based on the structure evolution proposed in Fig. 3d, there is no contribution of Pt-C species, which it is claimed to have in EXAFS data. Could the authors please elaborate where it comes from?
4. EXAFS spectrum of Pt1/Fe2O3-525 gives a clear signal of Pt-Fe bond as you marked on the graph – why is it not considered for fitting?
5. A very critical point is related to the XPS. Even though the spectra are of high interest and very insightful, the fitting of the spectra should be improved – at the moment there are not scientifically sound. Please, make sure that (i) the FWHM is similar in all Gaussian curves, (i) the fitting curve for each component (Pt 4f5/2 and Pt 4f7/2) touches the background, and (iii) correct splitting parameters are applied.
6. In view of the broad audience of the journal, the Reviewer believes that more explanation is required for the reason of adding pure Fe2O3 to the reaction mixture in the 9:1 ratio with respect to the catalyst. It is very important to exclude any catalytic contribution of pure Fe2O3 in the conditions applied, so it would make sense to show the activity of pure Fe2O3?
7. After each use of the catalyst, the time to get reasonable conversion slightly increases. How would you explain this trend? To exclude any leaching and single atoms agglomeration, ICP and XPS analyses of the catalyst after the 5th cycle should be provided. To prove the suggestion about the adsorption of organic components, TGA/DTA analysis could be very insightful.
Moreover, please consider some formatting changes to speed up the reader's understanding:
1. EDX mappings in Fig. S1 are too small and the signal is hardly visible. As well as scale bars in the HAADF-STEM. Increasing size will help a lot.
2. Figs S9-S11 are very valuable, however, the color of Pt is too similar to the color of Fe and the coordination sphere is not pronounced. Using a radically different color (green, yellow) for – also please consider color blinded people – is required. Additionally, the magnification of the structure will be beneficial.
3. Fig. S12 is not discussed in the manuscript.
4. In supplementary Information, line 43, the 30 ml/min is missing.

Reviewer #2 (Remarks to the Author):

This paper presents work concerning the production and use of single atom catalysts. Much of the

novelty in this paper relates to the method of production of the SAC. Indeed the methodology is remarkably simple but the key is the rapid heating and short dwell time. Longer dwell times result in nanoparticles. The authors then proceed to show that they have produced a SAC and that the temperature controls the nature of the SAC. The characterisation is convincing and the catalytic tests clearly show activity. The use of deuterium to probe the reaction is well considered and the DFT calculations are helpful in understanding the hydrogen/SAC interaction. The paper is well-written and easy to read.

Catalysts such as these have the potential to link homogeneous and heterogenous catalyst at the molecular level and help in our understanding of reaction mechanisms.

I recommend acceptance.

Reviewer #3 (Remarks to the Author):

Establishing the structure-performance relationship is quite important for heterogeneous catalysis. The authors show great efforts to minimize the gap between surface adsorption-reaction in heterogeneous catalysis and coordinate-reaction in homogeneous catalysis by using a special solid single-atom catalyst (SAC). The Pt SAC shows high activity and selectively in the hydrogenation of 3-nitrostyrene. The Pt-O coordination number and oxidation state of Pt is shown to correlate very well with the hydrogenation activity. I can support the publication after a minor revision, Please see my comments below:
1. It is interesting that ethanediamine ligands play a key role in the preparation of supported Pt SAC. Can the ligands be completely removed? I suggest that the authors supply more evidence on the negligible residues of the ligand.
2. The TOF value increases remarkably with decreased oxidation state of Pt. Notably, the catalyst with a Pt-O coordination of 2 exhibits an almost 2-3 times-high TOF value compared to the catalyst with a Pt-O coordination of 3. This result is very interesting. I suggest the authors putting more efforts on the discussion of this unusual increasing. Again, is it possible due to the continuous elimination of ethanediamine during thermal treatment? Or, would the geometric parameter, for example, the lattice constant and the Pt ensembles, show any effects on the activity?
3. Is the high selectivity unique for the SAC catalyst? I recommend the discussion.
4. The XPS for the reused catalyst is suggested to be provided.
5. The binding energy for Pt species shifts with the treating temperature. Can the author give the possible reason?
6. The authors contribute the activity loss to the organic compounds residue. I cannot fully agree. The residue on the active site would sometimes, significantly change its electronic structure, in this case, the Pt-O coordination and oxidized state. This fact may reduce the activity.

Responses to the referees

We are very grateful to the referees for their careful reading of the manuscript and raising many valuable comments which are definitely helpful to the improvement of the paper quality. The responses to each question or comment are shown below.

Referee 1

Authors report a novel method to prepare Pt single-atom catalysts with tunable coordination sphere aiming at establishing structure-performance relationship for the chemoselective hydrogenation of 3-nitrostyrene. A detailed characterization of active species with distinct Pt-O coordination numbers is provided with clear conclusions. The Reviewer thinks that this work is of high relevance and has the potential to be published in Nat Commun. However, many clarifications and additional experiments are needed to meet the high standards required in this authoritative journal. The points to be addressed before publications are the following.

Answer: We greatly appreciate this referee for the positive comments.

Q1*: After the preparation of the catalysts, how can authors be sure that the chlorine complex is removed, but not deposited on the catalyst? Even if the reaction between chloroplatinic acid and ethanediamine yields 100% (which the Reviewer assumes not true), the residual chlorine stays in the solution and potentially may be deposited on the* Fe_2O_3 *surface. Then, the peak around 2.5 Å shown in the EXAFS spectra (Fig. 3a) could correspond to Pt-Cl bond and gradually decrease with elevating the treatment temperature (please refer to: https://doi.org/10.1016/S0920-5861(98)00267-3; doi:10.1006/jcat.2002.3601). Therefore, the presence of chlorine can act as a poison for* Pt_1/Fe_2O_3*-500, -525 materials.*

Answer: In order to address this concern, we added the EXAFS spectra of H_2PtCl_6 to Fig. 3a. As you see, the Pt-Cl and Pt-O peaks are well separated, and the Pt-Cl contribution is absent in all our catalysts.

The absence of chlorine in the catalyst can also be justified by the synthesis process. In our synthesis of these series of catalysts, ethanediamine (1 ml, 15 mmol) was in a much excess amount compared to chloroplatinic acid (0.05 mmol), which corresponded to the molar ratio of $n_{ethanediamine}/n_{chloroplatinic\ acid}$ as high as 300 and led to the formation of a strong basic solution (pH = 12). In this case, all the H_2PtCl_6 should react with ethanediamine to give $(Pt(en)_2)^{2+}$ cations. Considering that the isoelectric point of the FeOOH support surface is around 6.5, the support surface should be negatively charged in a strong basic solution. Therefore, the positively charged $(Pt(en)_2)^{2+}$ cations will be preferentially adsorbed on the FeOOH surface, whereas the Cl^- anions will stay in the solution due to electrostatic repulsion.

Taken together, we can safely preclude either the existence of residual chlorine on the catalyst surface or the Pt-Cl contribution in the EXAFS spectra.

***Q2**: 1 min of keeping the sample in the furnace is not enough to reach thermal equilibrium due to the heat transfer and, thus, the samples actually were not heated up to reported temperatures- evidences are required in order to state that the set temperature was reached by the sample.*

Answer: This is a good question. In order to measure the temperature that was actually reached on the catalyst surface, we inserted the thermocouple into the powder sample during the RTT process. The sample temperature change was recorded and shown in the figure R1 below. It can be clearly seen that it took 40 ~ 50 s to reach the target temperature (500 – 600 ^{o}). In the revised manuscript, we have added Fig. R1 to the Supporting Information (Fig. S1) and mentioned it in the text (page 4, line 3 from bottom).

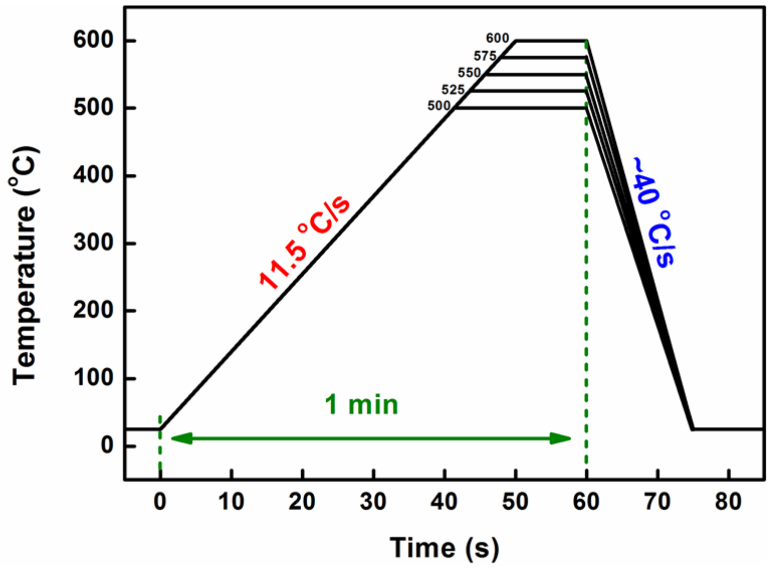

Fig. R1 The temperature profile of the sample during the RTT process.

***Q3**: Based on the structure evolution proposed in Fig. 3d, there is no contribution of Pt-C species, which it is claimed to have in EXAFS data. Could the authors please elaborate where it comes from?*

Answer: The contribution of Pt-C arises from the $(Pt(en)_2)^{2+}$ complex, as shown in Fig. 3d, and this contribution is located in the second shell of the central Pt atom. After adsorption of the $(Pt(en)_2)^{2+}$ complex on the FeOOH support, the Pt-C contribution still existed, but it was absent from the samples after the RTT at high temperatures owing to the decomposition of the en ligand. Therefore, in the EXAFS spectra (Fig. 3a), only the two samples, Pt-en (liquid) and the Pt_1/FeOOH-RT, present the Pt-C contributions at around 2.4 Å, which coincidently overlaps with the Pt-Pt

contribution.

However, for both Pt-en (liquid) and $Pt_1/FeOOH$-RT samples, O atom from H_2O and Fe atom from FeOOH may also be located in the second shell of Pt central atom, which would make it quite difficult to get reliable fitting results in the second shell. For this reason, we re-fitted the EXAFS data only based on the first shell contributions of the central platinum atom, that is, Pt-O and Pt-Fe contributions. The revised fitting result is shown in Table R1 below (corresponding to Table 1 in the revised manuscript). As you see, the trend of Pt-O CN with the RTT temperature is totally consistent with our previous version, but the exact figures look more reasonable and consistent with DFT result.

Table R1 The best-fitted structural parameters for Pt_1/Fe_2O_3-T catalysts as determined by EXAFS at the Pt L_{III} edge.[a]

Sample	Shell	CN	R (Å)	σ^2 (10^{-2} Å^2)	ΔE_0 (eV)	r-factor (%)
Pt Foil	Pt-Pt	12.0	2.76	0.5	4.9	0.001
PtO_2	Pt-O	6.0	1.99	0.4	3.2	0.3
H_2PtCl_6 (liquid)	Pt-Cl	6.0	2.32	0.4	-3.7	0.02
Pt-en (liquid)	Pt-N	4.3	2.01	0.5	5.7	0.3
$Pt_1/FeOOH$-RT	Pt-(N/O)	6.0	1.99	0.6	0.3	0.5
Pt_1/Fe_2O_3-500	Pt-O	3.8	2.00	0.7	4.5	0.6
Pt_1/Fe_2O_3-525	Pt-O	3.1	2.00	0.7	5.1	0.6
	Pt-Fe	0.2	2.63	0.4	5	
Pt_1/Fe_2O_3-550	Pt-O	2.8	2.00	0.7	4.9	0.6
	Pt-Fe	0.3	2.61	0.3	4.9	
Pt_1/Fe_2O_3-575	Pt-O	2.0	2.02	0.6	7	0.2
	Pt-Fe	0.7	2.62	0.3	7	
Pt_1/Fe_2O_3-600	Pt-O	1.8	2.02	0.6	6.5	0.5
	Pt-Fe	0.8	2.63	0.3	6.5	

[a] CN, the coordination number for the absorber-backscatterer pair. R, the average absorber−backscatterer distance. σ^2, the Debye−Waller factor. ΔE_0, the inner potential correction. The accuracies of the above parameters are estimated as CN, ±20%; R, ±1%; σ^2, ±20%; ΔE_0, ±20%. The data range used for data fitting in k-space (Δk) and R-space (ΔR) are 3.0-10.5 Å^{-1} and 1.2-3.2 Å, respectively.

***Q4**: EXAFS spectrum of Pt_1/Fe_2O_3-525 gives a clear signal of Pt-Fe bond as you marked on the graph – why is it not considered for fitting?*

Answer: Thanks for this kind reminding. Because the Pt-Fe bond contributed very minor, it was not considered in our previous version. In the revised manuscript, we have re-fitted the EXAFS data by considering the Pt-Fe contribution. As you see in Table R1, the Pt-Fe contribution is indeed minor (Pt-Fe CN is 0.2) in the Pt_1/Fe_2O_3-525.

Q5*: A very critical point is related to the XPS. Even though the spectra are of high interest and very insightful, the fitting of the spectra should be improved – at the moment there are not scientifically sound. Please, make sure that (i) the FWHM is similar in all Gaussian curves, (i) the fitting curve for each component (Pt 4f5/2 and Pt 4f7/2) touches the background, and (iii) correct splitting parameters are applied.*

Answer: Thank you for this instructive suggestion. In the revised manuscript, we have re-fitted the XPS by following the three rules that the reviewer mentioned above. The re-fitted XPS are shown Fig. R2 (corresponding to Fig. 3c in the revised manuscript), and the corresponding parameters are shown in Table R2 (corresponding to Table S1 in the revised SI).

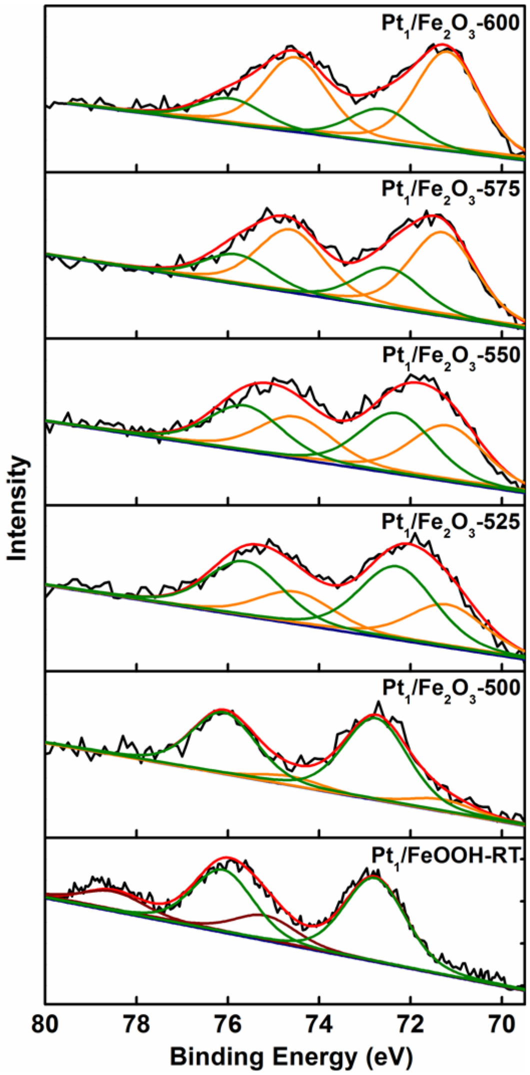

Fig. R2. The Pt 4f XPS spectra of Pt_1/Fe_2O_3-T catalysts.

Table R2 Pt 4f XPS spectra parameters of different Pt_1/Fe_2O_3-T catalysts

Sample	Binding energy of Pt $4f_{7/2}$ (eV)	Content (%)	FWHM (eV)	$\Delta(4f_{7/2}-4f_{5/2})$ (eV)	Average oxidation state
Pt_1/FeOOH-RT	75.18 (Pt^{IV})	24.0	1.57	3.33	2.48
	72.78 (Pt^{II})	76.0	1.57		
	- (Pt^{0})	-	-		
Pt_1/Fe_2O_3-500	- (Pt^{IV})	-	-	3.33	1.74
	72.76 (Pt^{II})	87.0	1.65		
	71.30 (Pt^{0})	13.0	1.65		
Pt_1/Fe_2O_3-525	- (Pt^{IV})	-	-	3.33	1.25
	72.30 (Pt^{II})	62.7	1.93		
	71.20 (Pt^{0})	37.3	1.93		
Pt_1/Fe_2O_3-550	- (Pt^{IV})	-	-	3.33	1.04
	72.30 (Pt^{II})	51.8	1.89		
	71.20 (Pt^{0})	48.2	1.89		
Pt_1/Fe_2O_3-575	- (Pt^{IV})	-	-	3.33	0.64
	72.48 (Pt^{II})	32.1	1.71		
	71.30 (Pt^{0})	67.9	1.71		
Pt_1/Fe_2O_3-600	- (Pt^{IV})	-	-	3.33	0.51
	72.62 (Pt^{II})	25.7	1.68		
	71.20 (Pt^{0})	74.3	1.68		

***Q6**: In view of the broad audience of the journal, the Reviewer believes that more explanation is required for the reason of adding pure Fe_2O_3 to the reaction mixture in the 9:1 ratio with respect to the catalyst. It is very important to exclude any catalytic contribution of pure Fe_2O_3 in the conditions applied, so it would make sense to show the activity of pure Fe_2O_3?*

Answer: Thank you for the valuable comment. As we have indicated in our previous work (*Nat. Commun. 5, 5634, 2014.*), pure Fe_3O_4 or Fe_2O_3 was inactive at all under identical reaction conditions to that for other catalysts. In the revised manuscript, we have added the reaction data of pure Fe_2O_3 in Table 2 and commented in the text.

The reason for adding 90 mg pure Fe_2O_3 to 10 mg catalyst in our reaction tests is to ensure the uniform dispersion of the catalyst in the reaction medium such that reproducible results could be obtained. It would cause testing errors if 10 mg sample is used without dilution with pure Fe_2O_3 support due to handling loss, in particular for the recycling tests.

***Q7**: After each use of the catalyst, the time to get reasonable conversion slightly increases. How would you explain this trend? To exclude any leaching and single atoms agglomeration, ICP and XPS analyses of the catalyst after the 5th cycle should*

be provided. To prove the suggestion about the adsorption of organic components, TGA/DTA analysis could be very insightful.

Answer: Thank you for this valuable comment. In fact, the slightly increase in the time to achieve the similar conversion during recycling tests (Table S3, corresponding to Table S4 in the revised SI) implies a slight deactivation of the catalyst due to partial covering of the active sites by adsorption of organic compounds. In order to exclude any leaching and single atoms aggregation, we performed ICP and XPS analysis of the catalyst after the 5th cycle. As shown in Table R3, the ICP analysis revealed the same Pt loading of the spent sample as the fresh one, indicating no leaching of Pt took place during the reaction. In agreement with the ICP analysis, the XPS results (also see Fig. R3, corresponding to Fig. S10 in the revised SI) showed that the spent catalyst has similar Pt/Fe ratio to the fresh one, and the Pt 4f binding energy almost unchanged after the reaction. All these results, in combination with the HAADF-STEM images (Fig. S9), have demonstrated that the chemical state of Pt single atoms did not change after the reaction.

From the TGA/DSC profile of the used catalyst (Fig. R4, corresponding to Fig. S8 in the revised SI)), one can clearly see that there is a weight loss of 1.5% at around 250 ^{o}C, indicating there is organic compounds covering the active sites, which led to the slight decrease of activity.

In the revised manuscript, we have added the above information and made the corresponding discussions.

Table R3 The ICP and XPS analysis results of the Pt_1/Fe_2O_3-600 and Pt_1/Fe_2O_3-600-used catalysts

Sample	Pt loading determined by ICP (%)	Pt/Fe surface atomic ratio determined by XPS (%)	Binding energy of Pt $4f_{7/2}$ (eV)
Pt_1/Fe_2O_3-600	0.9	1.92	71.32
Pt_1/Fe_2O_3-600-used	0.9	2.05	71.52

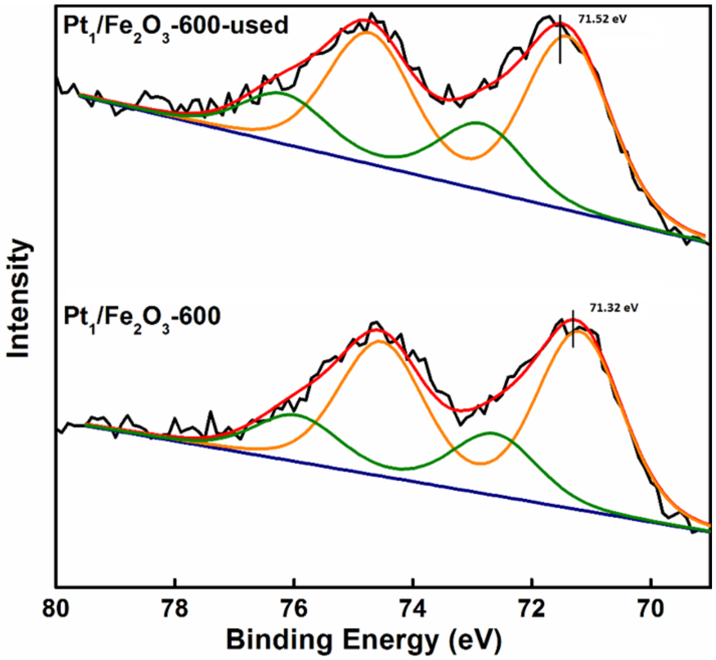

Fig. R3 Pt 4f XPS spectra of Pt_1/Fe_2O_3-600 and Pt_1/Fe_2O_3-600-used samples.

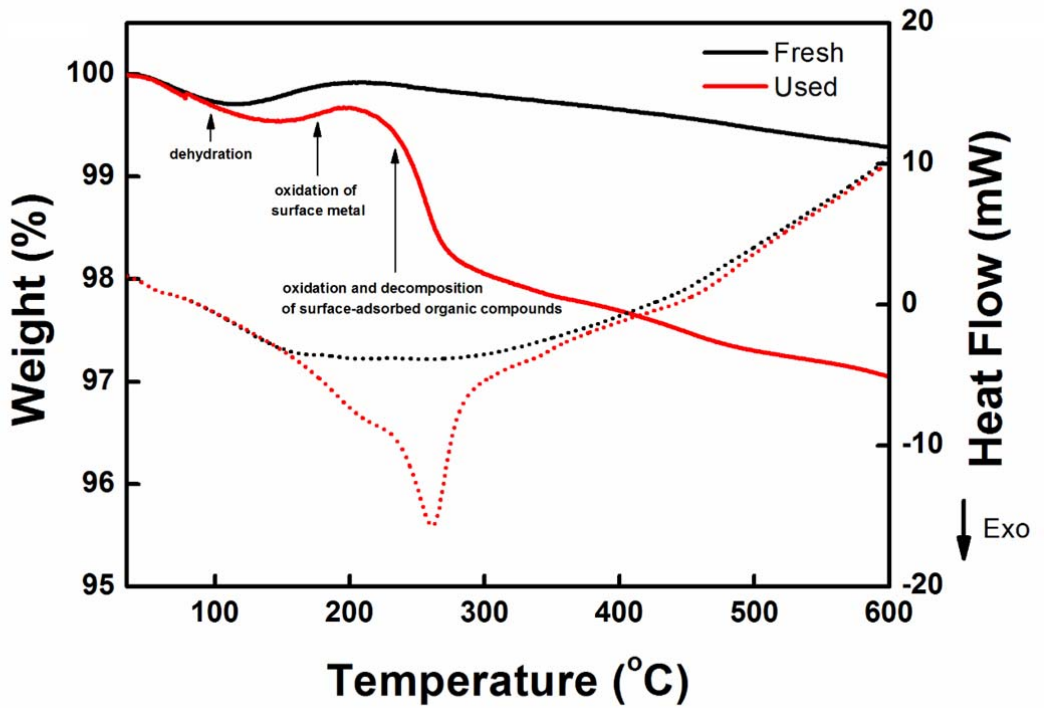

Fig. R4 The TGA/DSC profile of the Pt_1/Fe_2O_3-600-used sample in air. (TGA: solid line; DSC: dotted line)

***Q8**: Moreover, please consider some formatting changes to speed up the reader's understanding:*

1). EDX mappings in Fig. S1 are too small and the signal is hardly visible. As well as scale bars in the HAADF-STEM. Increasing size will help a lot.

Answer: Done.

2). Figs S9-S11 are very valuable, however, the color of Pt is too similar to the color of Fe and the coordination sphere is not pronounced. Using a radically different color (green, yellow) for – also please consider color blinded people – is required. Additionally, the magnification of the structure will be beneficial.

Answer: Done.

3). Fig. S12 is not discussed in the manuscript.

Answer: It has been mentioned in calculation of TOFs in the section of “methods”.

4). In supplementary Information, line 43, the 30 ml/min is missing.
Moreover, please consider some formatting changes to speed up the reader's understanding:

Answer: Thanks for your reminding. We have corrected this minor mistake.

Referee 2

This paper presents work concerning the production and use of single atom catalysts. Much of the novelty in this paper relates to the method of production of the SAC. Indeed the methodology is remarkably simple but the key is the rapid heating and short dwell time. Longer dwell times result in nanoparticles. The authors then proceed to show that they have produced a SAC and that the temperature controls the nature of the SAC. The characterisation is convincing and the catalytic tests clearly show activity. The use of deuterium to probe the reaction is well considered and the DFT calculations are helpful in understanding the hydrogen/SAC interaction. The paper is well-written and easy to read.

Catalysts such as these have the potential to link homogeneous and heterogenous catalyst at the molecular level and help in our understanding of reaction mechanisms.

I recommend acceptance.

Answer: We thank this referee for the positive comments.

Referee 3

Establishing the structure-performance relationship is quite important for heterogeneous catalysis. The authors show great efforts to minimize the gap between surface adsorption-reaction in heterogeneous catalysis and coordinate-reaction in coordination number and oxidation state of Pt is shown to correlate very well with the hydrogenation activity. I can support the publication after a minor revision, Please see my comments below:

***Q1**: It is interesting that ethanediamine ligands play a key role in the preparation of supported Pt SAC. Can the ligands be completely removed? I suggest that the authors supply more evidence on the negligible residues of the ligand.*

Answer: Thanks for this good question. In order to approve the en ligand was completely removed by RTT at high temperatures, we performed XPS analysis of the series of Pt_1/Fe_2O_3-T catalysts. As you see in the Table R4 below, the surface N/Fe ratio was at a pretty low level (0.02 ~ 0.04) for all the catalysts samples except for Pt_1/FeOOH-RT. In fact, such a low level of N species is similar to that of the ligand-free sample (H_2PtCl_6-ethylene glycol/Fe_2O_3-600 catalyst, N/Fe ratio was 0.036). This result, along with the EXAFS result, leading to the consistent conclusion that the en ligand was completely removed by RTT process. On the contrary, the Pt_1/FeOOH-RT sample which was not treated by RTT procedure, contained a much higher N species on the surface (N/Fe ratio was 0.079). It should be mentioned that our result was also in agreement with the literature report that Pt-N bond completely decomposed and transformed to metallic Pt upon treatment at 250 ^oC ~ 300 ^oC (*Thermochimica Acta, 1983, 71, 265.*)

Table R4 Surface N/Fe atom ratio of the different Pt/Fe_2O_3 catalysts.

Sample	Surface N/Fe atomic ratio
Pt_1/FeOOH-RT	0.079
Pt_1/Fe_2O_3-500	0.041
Pt_1/Fe_2O_3-525	0.040
Pt_1/Fe_2O_3-550	0.027
Pt_1/Fe_2O_3-575	0.026
Pt_1/Fe_2O_3-600	0.024
H_2PtCl_6/Fe_2O_3-600	0.026
H_2PtCl_6-ethylene glycol/Fe_2O_3-600	0.036

***Q2**: The TOF value increases remarkably with decreased oxidation state of Pt. Notably, the catalyst with a Pt-O coordination of 2 exhibits an almost 2-3 times-high TOF value compared to the catalyst with a Pt-O coordination of 3. This result is very interesting. I suggest the authors putting more efforts on the discussion of this unusual increasing. Again, is it possible due to the continuous elimination of ethanediamine*

during thermal treatment? Or, would the geometric parameter, for example, the lattice constant and the Pt ensembles, show any effects on the activity?

Answer: Thanks for this good question. In fact, the local coordination number of Pt-O affects the activity via changing the electronic state of Pt. As shown in Fig. 4, the lower is the coordination number of Pt-O, the lower is the average oxidation state of Pt single atoms, and then the higher is the hydrogenation activity. The underlying reason is the orbital interaction of Pt single atoms and hydrogen molecule. As shown by our DFT calculations (Fig. 5), when the Pt-O coordination number is 2, the hydrogen molecule can be easily activated without any barrier; in contrast, with an increase of the Pt-O coordination number, the barrier for hydrogen dissociation becomes higher, indicating hydrogen becomes more and more difficult to dissociate. This result is also in good agreement with our hydrogen chemisorption as well as kinetic isotope effect experimental results.

Finally, we again emphasize that the ethanediamine ligand was completely removed by our RTT procedure, and thus had nothing to do with the activity.

***Q3**: Is the high selectivity unique for the SAC catalyst? I recommend the discussion.*

Answer: Yes, the high selectivity is unique for SAC catalysts. For example, when the single atoms aggregated to NPs under certain conditions (For example, by RTT treatment at 600 ^{o}C for 10 min, the Pt/Fe_2O_3-600-10 catalyst), the selectivity dropped down from 98.0% to 63.1% (Table 2). The underlying reason for the uniqueness of the SACs for high chemoselectivity lies in the unique adsorption mode. When the Pt species is dispersed as single atoms, the substrate will adsorb via a top mode, that is, the nitro group is preferentially adsorbed through its two oxygen atoms on both Pt and the neighboring Fe or oxygen vacancy, thus leading to high chemoselectivity. In contrast, upon formation of Pt NPs, the substrate will be adsorbed on Pt NPs in a lying mode, that is, both the –C=C group and the benzene ring can be adsorbed on the catalyst surface, thus leading to the hydrogenation of –C=C bond, giving rise to low chemoselectivity. In the revised manuscript, we have discussed this uniqueness in more words.

***Q4**: The XPS for the reused catalyst is suggested to be provided.*

Answer: Thanks for this good suggestion. In the revised manuscript, we have provided the XPS analysis results of the reused catalyst (Table S4, Fig. S10, also see our answer to the referee #1). Compared with the fresh sample, there was no obvious change in either the surface Pt concentration or the electronic state of Pt, indicating excellent stability of the Pt_1/Fe_2O_3 catalysts.

***Q5**: The binding energy for Pt species shifts with the treating temperature. Can the author give the possible reason?*

Answer: We have elaborated this point in the manuscript. In brief, with the elevation of the RTT temperature, the local coordination number (Pt-O) of Pt single atoms became lower due to gradual removal of the neighboring oxygen atoms, which led to the increase of the electron density of Pt single atoms, and thus the down-shift of the binding energy.

Q6*: The authors contribute the activity loss to the organic compounds residue. I cannot fully agree. The residue on the active site would sometimes, significantly change its electronic structure, in this case, the Pt-O coordination and oxidized state. This fact may reduce the activity.*

Answer: To address this concern, we performed XPS (Table S4, Fig. S10), XAFS (Fig. S11) and TGA/DSC (Fig. S8) analyses of the used catalyst after the 5th cycle. As you see, neither the Pt-O coordination number nor the oxidation state was changed after the 5th cycle, indicating the chemical nature of the active sites did not change during the recycling tests. On the contrary, the TGA/DSC clearly showed that there was a weight loss of 1.5% at around 250 ^{o}C, which indicates that some organic species adsorbed on the catalyst surface and covered a part of the active sites, thus reduced the activity. Please also see our answer to referee #1.

REVIEWERS' COMMENTS:

Reviewer #1 (Remarks to the Author):

Authors have revised the manuscript accordingly, clarifying discussion and data are added. Thus, concerns of the Reviewer are addressed and the manuscript is recommended for publication in Nature Communications.

Reviewer #3 (Remarks to the Author):

The authors replied to my comment reasonably well. I have no further comments. The manuscript should now be acceptable by Nature Communications.
